# Toxicity of the Antiretrovirals Lamivudine, Dolutegravir, and Tenofovir on the Microalga *Chlorella vulgaris*

**DOI:** 10.1021/acsomega.6c00162

**Published:** 2026-06-01

**Authors:** Gabriel Souza-Silva, Mariângela Alcantara, Carolina Moreira, Maria Claras Starling, Cléssius Souza, Kenia Nunes, Cíntia Pereira, Marcos Mol, Micheline Silveira

**Affiliations:** † Postgraduate Program in Medicines and Pharmaceutical Assistance, School of Pharmacy, 28114 Federal University of Minas Gerais, Belo Horizonte 31270-901, MG, Brazil; ‡ Department of Research and Development, Ezequiel Dias Foundation, Department of Research and De-velopment, Belo Horizonte 30510-010, Minas Gerais, Brazil; § Department of Sanitary and Environmental Engineering, School of Engineering, 28114 Federal University of Minas Gerais, Belo Horizonte 31270-901, Minas Gerais, Brazil; ∥ Department of Biomedical Engineering and Science, Florida Institute of Technology, Melbourne, Florida 32901, United States; ⊥ Department of Parasitology, Institute of Biological Sciences, Federal University of Minas Gerais, Belo Horizonte 31270-901, Minas Gerais, Brazil

## Abstract

This study evaluated the toxicity of the three antiretrovirals: dolutegravir (DTG), tenofovir (TDF), and lamivudine (3TC), both as commercial medicines and active pharmaceutical ingredients (APIs), on the microalga *Chlorella vulgaris*. Acute (4-d) and chronic (14-d) toxicity tests were conducted following OECD Guideline 201 (2011). Among the evaluated substances, DTG-API showed the highest toxicity, while 3TC was the least toxic. Results indicate that excipients modulate toxicity and that chronic exposures may induce tolerance mechanisms in *C. vulgaris*. DTG-API was classified as “toxic,” with EC50 values of 3.3 mg/L (acute) and 5.9 mg/L (chronic). DTG-Med also showed acute toxicity (EC50 = 7.1 mg/L) but was almost nontoxic chronically. TDF-Med was considered “slightly toxic” acutely (EC50 = 92.9 mg/L) and “practically non-toxic” in chronic exposure. TDF-API showed higher chronic toxicity (EC50 = 88.7 mg/L) compared to its commercial form. 3TC-Med and 3TC-API were “practically non-toxic” under both conditions. Hormesis effects were observed at low concentrations of TDF-Med. Overall, APIs were more toxic than commercial medicines, suggesting a role of excipients in reducing toxicity. In addition, the Environmental Risk Assessment identified 3TC as the compound with the highest risk quotients, particularly in surface waters and wastewater treatment plant effluents, where medium to high ecological risks were observed (RQ > 7.0). In contrast, TDF presented insignificant risk across all evaluated environmental compartments (RQ < 0.01). Due to the limited availability of environmental occurrence data, a comprehensive risk assessment for DTG could not be performed, highlighting an important data gap in current monitoring studies. The results also highlight that conventional wastewater treatment processes may not be fully effective in mitigating these risks, reinforcing the need for improved monitoring programs and regulatory strategies to protect aquatic ecosystems and water resources.

## Graphical abstract

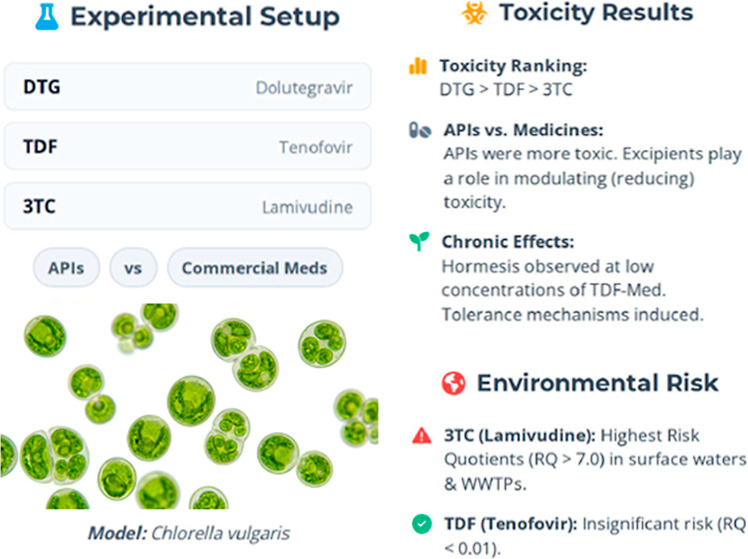

## 1 Introduction

Globally, more than 39 million people live with the human immunodeficiency virus (HIV).^1^ Consequently, currently, antiretroviral drugs (ARVs) rank among the most frequently prescribed pharmaceutical classes. In Brazil, approximately 900,000 people use these medicines on a daily and continuous basis,^2^ resulting in widespread distribution of pharmaceutical residues in the environment.^3–10^ Tenofovir (TDF) and lamivudine (3TC), both reverse transcriptase inhibitors, are among the most widely prescribed ARVs worldwide. Together with dolutegravir (DTG), they form the main treatment regimen used in Brazil since 2017,^2^ increasing the likelihood of their residues being detected in aquatic environments.

TDF and 3TC are administered at a daily dose of 300 mg each, while DTG, recently incorporated into HIV treatment, is prescribed at 50 mg/day. After administration, approximately 70–80% of TDF and 70% of 3TC are excreted unchanged in the urine, whereas about 53% of DTG is eliminated unchanged in the faeces.^11^ The high consumption of these drugs, combined with the inadequate removal capacity of wastewater treatment processes, results in their frequent detection in aquatic environments.^3–10^

Evidence is mounting that antiretroviral residues may alter physiological processes, behavioral patterns, and reproductive performance of aquatic species under environmentally relevant exposure levels.^12^ Therefore, assessing the ecotoxicological effects of ARVs in water bodies, as well as developing effective water treatment technologies for their removal, is crucial for environmental and public health.^13^

Understanding the toxic effects of ARVs residues on microalgae is essential to assess their impact on ecosystems and environmental balance. These organisms form the base of the aquatic food chain and are frequently used as key indicators for evaluating the ecological risks of pollutants.^14^ Previous studies suggest that oxidative stress and photosynthetic impairment are the main toxicity mechanisms of several pollutants in microalgae.^15–17^

The green microalga *Chlorella vulgaris* is a cosmopolitan species widely used as a biological model in ecotoxicological research due to its ecological relevance, rapid growth, and sensitivity to pollutants.^14^ As a primary producer, *C. vulgaris* plays a crucial role in nutrient cycling and oxygen production within aquatic ecosystems. This freshwater green microalga is widely used as a model organism in ecotoxicological studies due to its ecological relevance and sensitivity to a broad range of contaminants. Previous studies have demonstrated its effectiveness in assessing the toxicity of pharmaceuticals and other emerging pollutants.^18^ In addition, its use in standardized growth inhibition assays is recommended by the OECD Guideline 201 (2011), which supports its suitability for evaluating the environmental risks of contaminants, including antiretroviral residues.

Recently, we investigated the toxicity of the same antiretroviral compounds (TDF, 3TC, and DTG) in the cyanobacterium *Microcystis novacekii*, a prokaryotic photosynthetic organism.^19^ While cyanobacteria and green microalgae occupy similar ecological niches as primary producers, they differ fundamentally in cellular organization, metabolic pathways, and physiological responses to xenobiotics. Cyanobacteria are prokaryotes lacking membrane-bound organelles, whereas *C. vulgaris* is a eukaryotic microalga with compartmentalized cellular structures, distinct photosynthetic machinery, and potentially different detoxification and stress-response mechanisms.

Therefore, evaluating the same pharmaceutical residues in these two taxonomically and physiologically distinct groups enables a cross-taxonomic comparison that may reveal differential sensitivity patterns and improve understanding of how antiretroviral drugs impact primary producers at the base of aquatic food webs. Such comparative assessment is essential for refining environmental risk evaluations and avoiding extrapolations based solely on single-species data.

Thus, it is vital to explore the toxicity mechanisms of these residues in microalgae and to compare their toxicity patterns with previously reported responses in cyanobacteria, contributing to a broader understanding of cross-taxonomic sensitivity among aquatic primary producers and improving ecological risk assessment across different trophic levels. Therefore, this study aims to evaluate the toxicological effects of the ARVs TDF, 3TC, and DTG, both as commercial medicines and active pharmaceutical ingredients (API), on the freshwater microalga *C. vulgaris*, to understand the potential environmental impacts of these contaminants.

## 2 Materials and Methods

### 2.1 Medicines

The dolutegravir based formulation (DTG-Med) employed in this research contained 50 mg of the active pharmaceutical ingredient (DTG) along with excipients such as sodium stearyl fumarate, microcrystalline cellulose 101, povidone K30, mannitol 25 C, sodium starch glycolate, and the film coating Opadry II Yellow (composed of partially hydrolyzed poly­(vinyl alcohol), talc, titanium dioxide, macrogol/PEG, and yellow iron oxide). Each tablet weighed on average 307.3 ± 3.0 mg, with the active component representing roughly 16.2% of the total mass (manufacturer: Blanver Farmoquímica e Farmacêutica S.A., São Paulo, Brazil).

The tenofovir disoproxil fumarate formulation (TDF-Med) used in the assays provided 300 mg of the active ingredient (TDF) per tablet, together with sodium croscarmellose, lactose monohydrate, starch, microcrystalline cellulose, magnesium stearate, sodium lauryl sulfate, hypromellose, titanium dioxide, macrogol, and indigo carmine aluminum lake as colorant. Tablets had a mean weight of 687.1 ± 6.4 mg, in which the active drug accounted for approximately 43.7% of the composition (produced by Fundação Ezequiel Dias—FUNED, Minas Gerais, Brazil).

The lamivudine product (3TC-Med) contained 150 mg of lamivudine per unit, combined with magnesium stearate, silicon dioxide, sodium starch glycolate, microcrystalline cellulose, and a white Opadry YS-1-7003 coating (hypromellose, macrogol, titanium dioxide, and polysorbate 80). Each tablet had an average mass of 226.3 ± 2.6 mg, with the active substance corresponding to about 66.3% of its total weight (supplied by Fundação para o Remédio Popular—FURP, São Paulo, Brazil).

### 2.2 Active Pharmaceutical Ingredients

The active pharmaceutical ingredient dolutegravir (DTG-API), purity ≥95.0%, was purchased from Biosynth (Switzerland), through funding provided by the Research Support Foundation of the State of Minas Gerais (FAPEMIG) and the National Council for Scientific and Technological Development (CNPq).

The active pharmaceutical ingredient tenofovir disoproxil fumarate (TDF-API), purity ≥99.9%, was provided by the Ezequiel Dias Foundation (Funed) through its Industrial Directorate (DI), where it was analyzed in commercial medicines, identified, and certified for use in this study.

The active pharmaceutical ingredient lamivudine (3TC-API), purity ≥99.5%, was provided by the Faculty of Pharmacy of the Federal University of Minas Gerais (UFMG) through the Center for Pharmaceutical Analytical Development and Studies (CEDAFAR), where it was identified and standardized for the analyses conducted in this study. The physicochemical properties of these substances are described in Table 1.

**Table 1:**
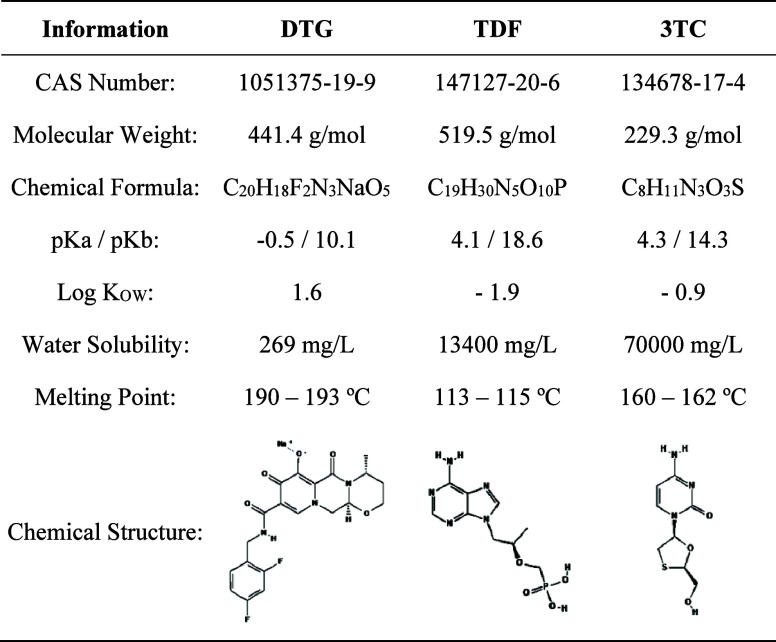
Physicochemical Characteristics of the Active Compounds Dolutegravir, Tenofovir Disoproxil Fumarate, and Lamivudine^a^

a Caption: TDF = tenofovir disoproxil fumarate; DTG = dolutegravir; 3TC = lamivudine; CAS = Chemical Abstracts Service; p*K*_a_ = logarithm of the acid dissociation constant; pKb = logarithm of the base dissociation constant; Log KOW = logarithm of the *n*-octanol–water partition coefficient.

### 2.3 Test Organism Culture

Cultures of *C. vulgaris* [Beijerinck] Beyerinck 1890 (isolated from water samples collected in Córrego da Mina—São Francisco River Basin (19°58′748″S; 43°49′259″W), Minas Gerais, Southeast Brazil) were used in the tests of this study. The cultures were maintained under controlled conditions in germination chambers at 22.0 °C ± 2 °C with a 12/12 h light/dark photoperiod and light intensity of 45 ± 5 μmol/m^2^/s in the Water Laboratory. The medium used for cultivation and testing of *C. vulgaris* was BG-11 (pH = 7.5 ± 0.2), supplemented with nitrogen (0.02 M Sodium nitrate—NaNO3).^20^

### 2.4 Preparation of Medicines

In this study, to ensure osmotic balance between the test organism and the test substances, all medicines and APIs were solubilized in BG-11 culture medium (with nitrogen). For the APIs 3TC and TDF, solubilization was performed in BG-11. For the DTG API, solubilization was carried out in BG-11 with the addition of dimethyl sulfoxide (DMSO) (Sigma-Aldrich, Germany) as a solvent at a maximum concentration of 0.1%.

For the delivery of these test theoretical concentrations, stock solutions of the medications were prepared using three DTG-Med tablets (50 mg each) dissolved in 1 L of culture medium, obtaining a stock solution of 150 mg/L; two TDF-Med tablets (300 mg each) dissolved in 1 L of culture medium, obtaining a stock solution of 600 mg/L; and four 3TC tablets (150 mg each) dissolved in 1 L of culture medium, obtaining a stock solution of 600 mg/L. For the active pharmaceutical ingredients, 150 ± 0.5 mg, 600 ± 0.5 mg, and 600 ± 0.5 mg of DTG-API, TDF-API, and 3TC-API, respectively, were weighed on an analytical balance and dissolved in 1 L of culture medium.

For solubilization, the medicines were manually powdered (entirely used for solubilization) using a mortar and pestle made of porcelain. The material was subsequently transferred to a glass beaker and dissolved with the aid of a heated magnetic stirrer (IKA RT 15, Staufen/Germany), maintained at a controlled temperature of 25 ± 1 °C, with continuous agitation at 500 rpm for 10 min. Each resulting solution was then passed through qualitative filter paper, and its pH was adjusted to 7.5 ± 0.2 using a 0.1 M sodium hydroxide (NaOH) solution. The final test concentrations of the antiretroviral compounds are summarized in Table 2.

**Table 2:** Nominal Test Concentrations (mg/L) Applied Equally to Active Pharmaceutical Ingredients (APIs) and Corresponding Medicines (Med) Based on Dolutegravir, Tenofovir Disoproxil Fumarate, and Lamivudine in Toxicity Assays with the Microalga *Chlorella vulgaris* ^a^

ARV	test concentration (mg/L)
DTG	100; 50; 40; 30; 20; 10; 1; 0.1 and 0.01
3TC	400; 300; 200; 100; 50; 25; 10; 5 and 1
TDF	400; 300; 200; 100; 50; 25; 10; 5 and 1

a Caption: ARV = antiretroviral; TDF = tenofovir disoproxil fumarate; DTG = dolutegravir; 3TC = lamivudine.

Initial screening assays were conducted prior to the main experiments to define the range of concentrations to be tested (Table 2). Concentrations were adjusted to remain within the solubility and stability limits of each compound and to maintain osmotic balance with the BG-11 culture medium. This approach ensured robust, reproducible, and environmentally relevant toxicity assessments for both acute and chronic exposures.

To determine the concentrations to be tested, initial screening assays were performed prior to the main experiments. These preliminary tests ensured that the chosen concentration ranges generated a complete dose–response profile, encompassing effects ranging from 0% to 100% (growth inhibition) or 0% to −100% (growth stimulation) or 100% to 200% (algicidal).

This is crucial for accurately validating the dose–response curves and obtaining reliable EC50 values. By encompassing the full spectrum of biological responses, the study eliminated the need for data extrapolation and ensured statistical robustness. Furthermore, concentrations were adjusted to remain within environmentally relevant solubility limits and to maintain osmotic equilibrium with the BG-11 culture medium, thus providing scientifically sound and reproducible conditions for assessing chronic toxicity.

### 2.5 Solvent Control

Due to the low solubility of DTG-API in water, the solvent DMSO was used for solubilization of this active pharmaceutical ingredient, as it is an efficient aprotic solvent commonly employed in pharmaceuticals and APIs for (eco)­toxicological investigations.^21^ Prior to exposure of the microalgae, toxicity tests with DMSO were conducted following OECD protocol No. 201 (2011),^22^ at concentrations ranging from 0.01 to 5.0% DMSO. After performing the experiments, no significant differences (*p* > 0.05) were observed between the negative control group, and the solvent control up to 1.0% DMSO. This excludes the possibility of solvent effects influencing toxicity results on microalgae at the 0.1% DMSO concentration used with DTG-API. These results support the use of DMSO at 0.1% in the toxicity assays, ensuring that no solvent-related effects interfered with the observed biological responses.

### 2.6 Toxicity Test

The growth inhibition test with *C. vulgaris* was performed following the OECD guideline No. 201 (2011),^22^ with adaptations in the exposure period to allow chronic assessment (14 days). Although OECD 201 commonly uses a 72 h exposure for acute tests, the guideline allows flexibility depending on the experimental objectives. In this study, a 4 day exposure was selected for acute testing to ensure that *C. vulgaris* completed multiple growth cycles, allowing more robust estimation of growth inhibition. The 14 day exposure for chronic assessment was chosen to evaluate longer-term effects, including potential adaptation, recovery, and delayed toxicity responses. Extended exposure durations also provide important information relevant for environmental fate and biodegradation studies, which commonly use 14 day assays to assess contaminant persistence and transformation.^18,19^

Control validity was ensured through the use of both negative (BG-11 medium) and positive controls (NaCl at 6.0 g/L), as recommended by OECD guideline No. 201. Additionally, solvent control tests confirmed that DMSO at 0.1% had no significant effect on algal growth (*p* > 0.05), validating its use for DTG solubilization. The combination of 4 day (acute) and 14 day (chronic) exposure periods enabled a more comprehensive assessment of toxicity, capturing both immediate and delayed biological responses under environmentally relevant conditions.

For the assay, 250 mL Erlenmeyer flasks containing the test concentrations were inoculated with *C. vulgaris* cultures to reach an initial density of 1 × 10^6^ cells/mL. The experimental units were maintained in triplicate at 22.0 ± 1.0 °C, under a 12 h light/12 h dark photoperiod and constant agitation, for 4 days in the acute test and 14 days in the chronic test. During exposure, cell growth was monitored spectrophotometrically at 695 nm, on day 0 (T0), day 4 (T4), and day 14 (T14),^18,19^ and cell density (cells/mL) was estimated from a calibration curve (*R* ^2^ = 0.9978) obtained by linear regression (eq 1) between optical density and microscopic cell counts. Cell density represents the number of algal cells per milliliter of culture, used to monitor growth over time. Algal biomass for each group (controls and treatments) was calculated from cell concentration per milliliter (*y*) and cell density (*X*) using eq 1 (*R* ^2^ = 0.9978)

1 y=(8×106)×X−174462

Mean growth rates and inhibition curves were constructed as a function of the concentrations of the tested compounds. The specific growth rate (μ) was calculated for both the 4 day and 14 day exposure intervals. Optical density of the cultures was measured with a spectrophotometer (Spectroquant, Merck Millipore, Darmstadt, Germany) at 695 nm, whereas cell density was determined microscopically (Nikon Eclipse E200, Tokyo, Japan) using a Neubauer hemocytometer.

Based on the optical density values, effects of the different ARVs on cell growth inhibition, stimulation, algicidal activity, and cellular hormesis were evaluated. In the cell growth inhibition assay, effects on the test organisms were assessed by measuring cell biomass over time. Growth inhibition was expressed as a percentage reduction relative to the control; values greater than 0 and up to 100% were considered growth inhibition.

For cell growth stimulation, effects on test organisms were also assessed by biomass measurement over time, similarly to the growth inhibition assay. Growth stimulation was expressed as a percentage reduction relative to the control; negative values (below 0) indicated growth stimulation, occurring at all or only higher concentrations.

The algicidal effect was defined as the capacity of the ARVs to cause irreversible death or direct lysis of *C. vulgaris* cells. Unlike growth inhibition, which represents a reversible reduction in biomass and is limited to 100%, algicidal effects occur when cell density decreases below the initial inoculum, resulting in inhibition percentages exceeding 100%. This approach allows clear differentiation between reversible growth inhibition and irreversible cellular death, providing ecologically relevant information on compound toxicity.

Finally, the hormesis effect was considered a biphasic response to ARVs, where low concentrations stimulated cell growth or activity, while higher concentrations caused cellular inhibition.

### 2.7 Metabolic Activity Assay

The metabolic activity assay was conducted using 3-(4,5-dimethylthiazol-2-yl)-2,5- diphenylformazan, thiazolyl blue (MTT) (Sigma-Aldrich, Germany) with the microalgae *C. vulgaris*, following the method described by Barmshuri et al. (2023)^23^ with modifications by Souza-Silva et al. (2025).^18^ MTT solutions (5.0 mg/mL) were prepared in BG-11 culture medium using vortexing (Phoenix Luferco Ltd.a—AP-59, Brazil) to solubilize the dye. The solution was filtered through a 0.20 μm filter (PVDF), and all preparation was performed in the dark.

Microalgae cultures were treated with both active pharmaceutical ingredients (APIs) and their commercial formulations at concentrations corresponding to the no observed effect level (NOEC)—specifically 10 mg/L for TDF, 100 mg/L for 3TC, and 0.1 mg/L for DTG—for periods of 4 and 14 days under the same experimental conditions as the growth inhibition test. After the exposure phase, 1.0 mL samples from each treatment group were collected in triplicate and placed into 1.5 mL microtubes, to which 50 μL of MTT solution was added. The tubes were then incubated in the dark at 37 °C for 4 h. Following incubation, samples were centrifuged at 10,000 rpm for 5 min at ambient temperature, and the supernatant was carefully removed.

Subsequently, 200 μL of DMSO was added to the pellets in the microtubes, followed by incubation for 5 min at room temperature. The samples were then vortexed vigorously for 10 s, centrifuged at 10,000 rpm for 10 min under the same temperature conditions, and the resulting supernatants were transferred to a 96-well microplate (Thermo Scientific Multiskan FC, USA) for absorbance measurement at 570 nm. The blank control consisted of culture medium (BG-11) combined with the reagents (MTT and DMSO) in the absence of microalgal cells.

### 2.8 Environmental Risk Classification

Ecotoxicological assessments with algal species generally report aquatic toxicity as EC50 values obtained from 3- or 4 day exposure experiments. In contrast, information on chronic toxicity remains scarce, and testing methodologies for long-term effects lack full standardization, which complicates the classification of chronic risks. Therefore, in this study, the GHS was applied not to address these limitations, but to provide a standardized framework for comparing the intrinsic aquatic toxicity of the tested compounds based on acute end points. To evaluate the impact of ARVs on *C. vulgaris* in this study, the classification “Hazardous to the Aquatic Environment” outlined in Annex 2.28­(b) of the Globally Harmonized System of Classification and Labeling of Chemicals (GHS)^24^ was applied (see Table 3).

**Table 3:** Risk Categorization was Performed Using EC50 Values Obtained from the Ecotoxicological Tests with Microalgae, Following the Criteria Outlined in Annex 2.28­(b) of the Globally Harmonized System of Classification and Labelling of Chemicals (GHS)

EC50 value (*X*)	toxicity classification
*X* ≤ 1.0 mg/L	very toxic
1.0 < *X* ≤ 10.0 mg/L	toxic
10.0 < *X* ≤ 100.0 mg/L	slightly toxic
*X* > 100.0 mg/L	practically nontoxic

The use of GHS classification in this study was intended as a complementary framework to interpret EC50 values obtained under controlled experimental conditions (Tables S1–S3). In this context, GHS enables a consistent and internationally recognized comparison of hazard levels across compounds, based primarily on acute toxicity data. However, it does not account for chronic toxicity or long-term ecological effects and is therefore used here as a complementary tool rather than a substitute for comprehensive risk assessment. The GHS classification was applied to provide a standardized framework for comparing the intrinsic aquatic toxicity of the tested compounds based on EC50 values, allowing consistent ranking of their hazard potential.

### 2.9 Environmental Risk Assessment

The environmental risk assessment (ERA) was conducted using the ERA tool.^25^ In this study, the analysis was based on the calculation of Risk Quotients (RQs) (eq 2), defined as the ratio between the measured environmental concentration (MEC) and the predicted no-effect concentration (PNEC) for the antiretrovirals Tenofovir, Lamivudine, and Dolutegravir. Data on environmental levels of these compounds were retrieved from the PHARMS-UBA database.^26^

2 RQs=MEC/PNEC

The ecological risk of each target substance was classified into four levels based on RQ values: (i) RQ < 0.01: insignificant risk; (ii) 0.01 ≤ RQ < 0.1: low risk; (iii) 0.1 ≤ RQ < 1: medium risk; and (iv) RQ ≥ 1: high risk. The PNEC was defined as EC50 values (from the literature and/or obtained in the present study) divided by an assessment factor of 1000 for each organism. An assessment factor was applied to ensure a conservative safety margin when estimating the environmental effects of ARVs based on experimental data. This approach accounts for uncertainties associated with extrapolation from acute to chronic effects, interspecies variability, and differences between laboratory conditions and the natural environment, as well as potential unforeseen adverse effects.

### 2.10 Statistical Analysis

Statistical analyses were conducted using R software (version 4.5.0). Data distribution was first examined with the Shapiro–Wilk test to verify normality. Subsequently, results from each treatment were compared with the negative control group through nonparametric variance analysis (Kruskal–Wallis test). Differences were considered significant when *p*-values were below 0.05, corresponding to a 95% confidence level. Dose–response models, including log-logistic, log–normal, and Weibull functions, were evaluated with the “drc” package in R to identify the curve providing the fit.^27^ Model selection was based on the Akaike Information Criterion (AIC), with the lowest AIC indicating the best fit. These regression models describe the relationship between the independent variable (concentration or dose) and the dependent variable (biological response).

In addition to univariate analyses, a Principal Component Analysis (PCA) was conducted to integrate the different ecotoxicological parameters obtained (EC50 values from acute and chronic exposures, and variation in metabolic activity). This multivariate approach enabled the identification of overall toxicity patterns and the grouping of treatments according to their ecotoxicological profiles. PCA was conducted in R softwere using a standardized data matrix (z-score) and the “FactoMineR” package. The first two principal components were retained based on the Kaiser criterion (eigenvalues >1.0) and cumulative explained variance, enabling an integrated interpretation of the results.

Control validity was assessed according to the criteria established by the OECD 201.^22^ The test was considered valid when exponential growth was maintained in the control group throughout the exposure period, with a sufficient increase in biomass. In this study, the coefficient of variation of the average specific growth rate in control replicates did not exceed 30%, and the CV of daily growth rates did not exceed 5%, in accordance with guideline recommendations. All experiments fulfilled these validity criteria, indicating that the test conditions were appropriate and that the derived EC50 values are reliable and not affected by variability or impaired growth in the control.

## 3 Results and Discussion

### 3.1 Toxicity of the Antiretroviral Dolutegravir

In the present study, commercial formulations were tested as whole systems, including excipients, following dissolution and filtration procedures. This approach enhances environmental relevance, as pharmaceuticals enter aquatic environments primarily in their formulated forms. Excipients such as surfactants, binders, and coatings may alter physicochemical properties, including solubility and bioavailability, thereby influencing toxicity. Consequently, differences observed between Med and API forms suggest that risk assessments based solely on APIs may underestimate real-world environmental effects.^18,19^

Among the tested ARVs, DTG was the one that showed the highest toxicity to the microalga *C. vulgaris*. Table S1 presents the main results and observed effects of the toxicity of the active pharmaceutical ingredients and the commercial medicines based on DTG on the microalga.

It was observed that the estimated EC50 value for acute exposure to DTG-Med was 7.1 ± 0.1 mg/L, indicating that this ARV can be classified as “toxic” to the microalga *C. vulgaris*. For DTG-API, the observed toxicity to the microalga was even higher, with an estimated EC50 value of 3.3 ± 0.2 mg/L for acute exposure, highlighting a key role of excipients in the observed toxicity results. Like DTG-Med, DTG-API can be classified as “toxic” to the microalga under acute exposure.

However, it was not possible to estimate an EC50 value for chronic exposure to DTG-Med, as the highest concentration tested (100.0 mg/L) inhibited only 6.4% of cell growth, indicating very low apparent toxicity under chronic exposure conditions. It is important to note that the GHS-based classification thresholds were applied here only for comparative purposes and do not represent a formal classification of chronic toxicity. In contrast, the toxicity of DTG-API after 14-d of exposure remained high, with an estimated EC50 value of 5.9 ± 0.5 mg/L, which falls within the “toxic” range based on the applied thresholds, again used solely for comparative interpretation, to the microalga under chronic exposure.

Considering that exposure time directly influenced the response observed for DTG-Med, the increased exposure duration led to a significant reduction (*p* < 0.05) in toxicity. Therefore, *C. vulgaris* showed an apparent recovery in biomass at concentrations up to 20.0 mg/L, where no significant difference was observed between the treatment and control groups (*p* > 0.05). However, this response likely reflects population regrowth and generational turnover rather than true physiological recovery of the initially exposed cells, given the short generation time of the species.^14,19^

The lack of significant difference between control and treatment groups (*p* > 0.05) at concentrations up to 20.0 mg/L suggests that tolerance and/or physiological adaptation occurs within this range, as also observed in another study with levofloxacin.^28^ This indicates a concentration threshold below which *C. vulgaris* can mitigate the toxic effects of DTG-Med.

The increase in exposure time resulted in a significant reduction in toxicity (*p* < 0.05). This contrasts with organisms like *M. novacekii*,^23^ which show increased toxicity over time, suggesting that *C. vulgaris* has more efficient adaptive responses to DTG. However, this recovery behavior was drastically reduced during exposure to DTG-API, as chronic toxicity (EC50 = 5.9 mg/L) was only slightly lower than acute toxicity (EC50 = 3.3 mg/L), but still within a concerning range.

Although DTG-Med is classified as “toxic” under acute exposure (EC50 < 10.0 mg/L), it is considered “practically nontoxic” under chronic exposure (EC50 > 100.0 mg/L). This discrepancy may indicate that the acute toxic impact is related to transient effects that do not result in cumulative damage over time.^29^ While *C. vulgaris* can recover at concentrations up to 20.0 mg/L, the data suggest that recovery may be less effective at concentrations closer to 100.0 mg/L, where slight but significant effects on cell growth were observed.

Furthermore, the high toxicity observed, both acute and chronic, for DTG-API raises concerns regarding its true environmental toxicity. During treatment processes, especially filtration and adsorption, excipients in commercial medicines can be partially or completely removed, resulting in the release of the active ingredient in its purest form.^30^ This transformation can significantly alter the toxicological profile of the compound, increasing its bioavailability and potential toxicity to aquatic organisms, as seen in the DTG toxicity results. Thus, the final treated effluent may contain more reactive or bioavailable forms of the medicine, contributing to more severe environmental impacts.

In addition to these observed effects, both DTG-Med and DTG-API could cause algicidal effects on the microalga. Acute exposure of *C. vulgaris* to DTG-Med led to cell death with an estimated EC50 of 68.8 ± 3.8 mg/L. However, in chronic exposures, the algicidal effect was significantly reduced (*p* < 0.05), as the highest concentration tested (100.0 mg/L) resulted in an algicidal effect of only 12.5% of algal biomass.

On the other hand, acute exposure to DTG-API caused a greater algicidal effect than DTG-Med, with an estimated EC50 of 28.2 ± 4.4 mg/L, about 2.5 times more toxic than the commercial medicines. Additionally, chronic exposure also led to algicidal effects, with an EC50 of 36.2 ± 2.2 mg/L.

The results demonstrate that both DTG-Med and DTG-API exhibit algicidal effects on *C. vulgaris*, highlighting the toxic potential of this antiretroviral to aquatic photosynthetic organisms. Acute exposure to DTG-Med resulted in an EC50 of 68.8 ± 3.8 mg/L, indicating low toxicity. However, during chronic exposure, a significant reduction in toxicity was observed, with the highest tested concentration (100.0 mg/L) causing only 12.5% inhibition of biomass, possibly due to cellular adaptation mechanisms or partial degradation of the compound over time.

In contrast, DTG-API showed higher algicidal toxicity compared to commercial medicine, with an acute EC50 of 28.2 ± 4.4 mg/L, suggesting that excipients in the medicines may partially mitigate the toxic effects of the pure active ingredient. Nevertheless, DTG-API maintained its toxicity even under chronic conditions, with an EC50 of 36.2 ± 2.2 mg/L, indicating that the pure API presents a persistent and potentially more harmful effect on phytoplankton communities.

When evaluating the impact of DTG-Med exposure on the metabolic activity of *C. vulgaris*, it was observed that although concentrations of 0.1 mg/L did not inhibit its growth, there was a 22% reduction in metabolic activity compared to the control. For DTG-API, this effect was even greater, with a 44% reduction in metabolic activity compared to the control.

These results are consistent with previous findings, where exposure of *M. novacekii* to DTG-API led to a 56% reduction in metabolic activity at 0.1 mg/L of the antiretroviral.^23^ In both cases, DTG likely caused oxidative stress and/or chloroplast dysfunctions, affecting energy production without immediately compromising cell proliferation. Moreover, the reduction in metabolic activity may reflect an adaptive response by the microalga. This outcome indicates the presence of sublethal effects, possibly linked to interference by the medicine, or its excipients, in essential physiological processes such as photosynthesis, cellular respiration, or protein synthesis, as also observed for other pharmaceutical residues like antibiotics.^31^

### 3.2 Toxicity of the Antiretroviral Tenofovir

In the present study, the toxicity of TDF demonstrated distinct effects between the different forms of the medicine (TDF-Med and TDF-API) and between exposure durations (acute and chronic), highlighting not only variations in toxicity but also the occurrence of nonlinear responses, such as stimulation of algal growth at low concentrations of the medicine. These findings are relevant for understanding the potential ecological impacts of TDF, especially considering its environmental persistence and possible influence on phytoplankton communities. Table S2 presents the main toxicity results and the corresponding observed effects on the microalga.

The TDF-Med showed an estimated EC50 value of 92.9 ± 2.7 mg/L for acute exposure, indicating that this ARV can be classified as “slightly toxic” to the microalga *C. vulgaris*. For acute exposure, although cell inhibition was observed at higher concentrations of the medicine, at low concentrations (5.0 mg/L), a stimulatory effect on cell growth was noted, suggesting a possible hormesis effect. However, it was not possible to estimate the EC50 for cell inhibition after chronic exposure, as the highest tested concentration (400.0 mg/L) did not inhibit cell growth.

Additionally, even at low concentrations (5.0 mg/L), a significant increase in cell growth was observed compared to the control, indicating that this ARV may stimulate microalgal growth over time. Although the acute EC50 value (92.9 ± 2.7 mg/L) classifies TDF as “slightly toxic” to *C. vulgaris*. The absence of chronic cell inhibition highlights that the microalga displays tolerance and/or prolonged adaptation to the medicine.^32^

When assessing chronic cell growth of *C. vulgaris*, an EC50 value of 28.4 ± 3.3 mg/L was established for growth stimulation by TDF-Med. Although growth inhibition is the most used parameter in algal toxicity assays, the evaluation of growth stimulation is also extremely important.^33^ In this study, TDF-Med may act as a metabolic stimulator, promoting algal growth beyond that of the control. This phenomenon can mask adverse toxic effects and lead to misinterpretation of the compound’s safety.

Therefore, although no chronic cell inhibition was observed, the stimulation detected does not rule out the possibility that TDF-Med causes environmental toxic effects. The excessive growth of certain microalgae species, such as *C. vulgaris*, can disrupt aquatic ecosystems, contributing to undesirable blooms and eutrophication.^34^ Hence, considering both inhibition and stimulation of algal growth provides a more comprehensive understanding of the potential ecological impacts of emerging contaminants like pharmaceutical residues.

Moreover, the acute EC50 value (92.9 ± 2.7 mg/L) suggests that the toxic impact is more pronounced under short-term and high concentration conditions, which may be relevant in cases of point source pollution, such as discharges from industrial or hospital settings.^35–37^ The response of *C. vulgaris* to TDF-Med exposure is consistent with observations in other aquatic organisms, such as *M. novacekii*,^23^ *Synechococcus elongatus*, *Chlorococcum infusionum*,^38^ and *Biomphalaria glabrata*,^12^ which also showed low chronic toxicity to the same medicine.

The ability of *C. vulgaris* to withstand chronic exposure to TDF may be associated with the efficiency of its metabolic mechanisms in dealing with chemical stressors. Another factor is the alga’s potential to metabolize and/or excrete the medicine, reducing its toxic effects over time, which makes it a potential model for TDF biodegradation.^39–41^ The observed growth stimulation at low concentrations (5.0 mg/L) highlights a nonlinear dose–response behavior, where subinhibitory concentrations can stimulate physiological and metabolic processes. This low-dose effect may be attributed to an initial adaptive stimulus, where the presence of TDF at nonlethal levels activates cellular defense mechanisms such as antioxidant production or increased metabolic efficiency.^42,43^

In contrast, for acute exposure to TDF-API, no hormesis-like effect was observed, as was seen with the commercial medicine. The EC50 was estimated at 110.3 ± 5.3 mg/L for acute exposure, significantly higher than that observed for TDF-Med, while the EC50 was estimated at 88.7 ± 9.8 mg/L after 14-d of exposure. Thus, for TDF-API, toxicity was significantly greater after chronic exposure. These data indicate a substantial decrease in EC50 values over time, demonstrating that TDF-API exhibited greater toxicity under chronic conditions compared to acute exposure.

The absence of cell growth stimulation after chronic exposure, as observed with TDF-Med, suggests that excipients in the commercial medicine may play a key role in modulating toxicity and the biological response of the microalga.^44–46^ In the case of TDF-API, the microalga did not exhibit growth stimulation at low concentrations, possibly due to the absence of synergistic or modulating compounds present in the commercial medicine.

The higher toxicity observed in chronic exposure to TDF-API may be related to gradual bioaccumulation of the active pharmaceutical ingredient in the algal cells. Unlike TDF-Med, TDF-API may interact directly with metabolic and cellular systems without interference from other medicine components, resulting in more pronounced toxic effects over time.^14,47^ These effects may include inhibition of essential enzymatic mechanisms, damage to cell membranes, and impairment of photosynthesis, as reported in similar studies on pharmaceutical toxicity in microalgae. Moreover, the differing behavior between the commercial medicine and isolated API highlights the complexity of interactions between excipients and active compounds in pharmaceutical medicines.^48,49^

Although classified as “slightly toxic” or “practically non-toxic,” exposure of *C. vulgaris* to TDF-Med significantly altered cellular metabolic activity. Concentrations of 10.0 mg/L were sufficient to cause a 38% reduction in metabolic activity compared to the control (*p* < 0.05), whereas for TDF-API, no such effect was observed, with a nonsignificant 0.5% reduction (*p* > 0.05). For the cyanobacterium *M. novacekii*, exposure to TDF-API led to a 12% reduction in metabolic activity.^23^

However, in the present study, this effect was not observed in the microalga *C. vulgaris*. The difference in observed effects may be attributed to physiological, biochemical, and structural differences between these organisms. Cyanobacteria, being prokaryotes, have cellular characteristics that differ from eukaryotic microalgae, such as a peptidoglycan based cell wall and lack of intracellular compartmentalization, which may facilitate greater absorption and sensitivity to the medicine.^50^

### 3.3 Toxicity of the Antiretroviral Lamivudine

In general, the results indicated low toxicity of 3TC to the microalga *C. vulgaris*, with high EC50 values and minimal sublethal effects, even at high concentrations. Additionally, stimulation of cell growth was observed under some chronic exposure conditions. These findings reinforce the importance of considering sublethal and adaptive responses in ecotoxicological studies, especially in the context of pharmaceuticals with low direct toxicity but with the potential to alter the growth dynamics of photosynthetic organisms. Table S3 presents the main toxicity results and the respective effects observed on the microalga.

The 3TC-Med presented estimated EC50 values of 118.8 ± 4.2 mg/L for acute exposure and 209.6 ± 24.6 mg/L for chronic exposure, indicating that this ARV can be classified as “practically nontoxic” to the microalga *C. vulgaris* in both cases. Similarly, 3TC-API showed no toxicity to *C. vulgaris* after either acute or chronic exposure, with EC50 values exceeding 400.0 mg/L. Additionally, the highest concentration tested (400.0 mg/L) inhibited only 4.5% of cell growth (*p* < 0.05), while other concentrations showed no significant inhibition (*p* > 0.05) when compared to the negative control (0.0 mg/L).

However, after 14-d of chronic exposure of the microalga to 3TC-API, a significant increase in algal growth (*p* < 0.05) was observed at all treatment concentrations compared to the negative control. Thus, *C. vulgaris* exhibited growth stimulation in the presence of 3TC-API, with an EC50 value of 155.0 ± 8.5 mg/L after 14 days of exposure. This response may suggest a potential utilization or metabolic interaction with the compound; however, this interpretation is based on the results of the present study and should be considered with caution. Additionally, changes in concentration over time due to biological uptake or transformation cannot be excluded, which represents an inherent limitation of nominal exposure conditions. Moreover, *C. vulgaris* demonstrated an ability to recover from 3TC-API intoxication. During acute exposure, 200.0 mg/L of 3TC inhibited 83.1% of cell growth, while the same concentration under chronic exposure inhibited only 37.2%, representing a 2-fold reduction in the final inhibitory effect.

Additionally, after 4-d of exposure, concentrations above 10.0 mg/L of 3TC-Med significantly inhibited cell growth (*p* < 0.05), whereas after 14-d of exposure, only concentrations above 100.0 mg/L showed this effect (*p* < 0.05), representing a 10-fold reduction in sensitivity over time. Toxicity analysis of 3TC on *C. vulgaris* revealed important findings that suggest a concentration, and time dependent toxicological behavior. The pronounced inhibitory effect indicates that the medicine may be relatively more toxic during short-term exposure, suggesting *C. vulgaris* is particularly sensitive to 3TC over brief exposure periods.

This short vs long-term effect is further evidenced by comparing the concentration thresholds needed to cause significant growth inhibition, as also observed with other pharmaceutical residues.^51^ In acute exposure, concentrations above 10.0 mg/L showed significant effects (*p* < 0.05), while in chronic exposure, only concentrations above 100.0 mg/L differed significantly from the control (*p* < 0.05). This 10-fold difference suggests *C. vulgaris* possesses adaptation mechanisms that mitigate the medicine’s toxic effects over longer periods.^52^ The reduction in inhibition under chronic exposure reflects a possible decrease in algal responsiveness over time.

These results indicate that 3TC toxicity to *C. vulgaris* depends on both concentration and duration of exposure, with more pronounced effects in short-term scenarios. The greater acute toxicity could be attributed to a rapid and intense cellular response to the medicine, whereas the adaptation observed during chronic exposure may reflect the alga’s ability to develop resistance mechanisms over time.^53–55^ Additionally, the lower toxicity under chronic exposure may be related to the alga’s cellular repair or regeneration abilities. After 14-d, much higher concentrations (above 100.0 mg/L) were required to inhibit growth significantly, suggesting greater resilience in environments with constant medicine levels.

These findings are consistent with other studies showing that chronic medicine exposure can alter toxic responses in aquatic organisms, often resulting in less acute effects over time.^38,46^ Furthermore, comparisons with other medicines and active ingredients, such as the valsartan (VAL) study by Turek et al. (2023),^46^ demonstrate that excipients may play a crucial role in modulating toxicity and should be considered in environmental pharmaceutical risk assessments.

In contrast to 3TC-API, 3TC-Med, which contains excipients alongside the active ingredient, showed significantly lower EC50 values than the pure compound. The medicine exhibited greater toxicity in both acute and chronic exposures. This suggests that excipients may increase the bioavailability of the medicine or alter its physicochemical properties, leading to more harmful effects on aquatic organisms.^56–58^

The difference in toxicity between the API and the commercial medicine may be explained by the interaction of excipients with aquatic organisms. As noted by Turek et al. (2023),^46^ excipients like microcrystalline cellulose, magnesium stearate, and other components in the commercial 3TC medicine may have synergistic effects that enhance the toxicity of the active ingredient. Among the ARVs tested, both 3TC-Med and 3TC-API showed the lowest impact on the metabolic activity of *C. vulgaris*. Concentrations up to 100.0 mg/L did not alter the microalga’s metabolic activity, corroborating findings for the *M. novacekii*.^23^

This low toxicity may be linked to the medicine’s specific mechanism of action. 3TC is a nucleoside analog that inhibits reverse transcriptase. However, photosynthetic organisms like microalgae and cyanobacteria do not possess reverse transcriptase, as their replication does not involve reverse transcription. Therefore, the medicine likely does not interfere with central metabolic processes in these organisms, which explains the absence of significant toxic effects even at high concentrations.^59^

### 3.4 Toxicity of the Antiretroviral on *C. vulgaris*

Table 4 presents a comparative summary of the main ecotoxicological parameters evaluated in this study, including acute and chronic EC50 values, changes in metabolic activity (MTT assay). The integrated comparison between the pharmaceutical medicines and their respective active pharmaceutical ingredients (APIs) clearly illustrates the differences in toxicity between the commercial and pure forms of the compounds, as well as highlights sublethal effects associated with some of the tested ARVs.^60,61^

**Table 4:** Summary of the Ecotoxicological Parameters of the Antiretrovirals Dolutegravir (DTG), Tenofovir (TDF), and Lamivudine (3TC), in their Commercial Medicine (Med) and Active Pharmaceutical Ingredient (API) Forms, on the Microalga *C. vulgaris* ^a^

ARV	type	EC50 (4-d)	EC50 (14-d)	metabolic activity assay*
DTG	Med	7.1 ± 0.1 mg/L	>100.0 mg/L	- 22%
DTG	API	3.3 ± 0.2 mg/L	5.9 ± 0.5 mg/L	- 44%
TDF	Med	92.9 ± 2.7 mg/L	>400.0 mg/L	- 38%
TDF	API	110.3 ± 5.3 mg/L	88.7 ± 9.8 mg/L	- 0,5%
3TC	Med	118.8 ± 4.2 mg/L	209.6 ± 24.6 mg/L	n.o
3TC	API	>400.0 mg/L	>400.0 mg/L	n.o

a Caption: DTG = dolutegravir; TDF = tenofovir disoproxil fumarate; 3TC = lamivudine; Med = commercial medicine; API = active pharmaceutical ingredient; 3TC-Med = commercial medicine based on lamivudine; EC50 = concentration of effect (growth inhibition) on 50% of organisms; n.o = not observed. The notation “not observed” indicates that the specific target effect, whether Growth Inhibition, Growth Stimulation, or Algaecide, was not elicited by the compound within the tested concentration range, i.e., the substance did not produce the intended effect at any of the concentrations evaluated. * = Metabolic activity (% of control) determined by the MTT assay, expressed as the percentage of MTT reduction in treated cells relative to the negative control (considered as 100%).

The integrated comparison of the ecotoxicological parameters evaluated (Table 4) highlights marked differences in the toxicity of the antiretrovirals DTG, TDF, and 3TC on the microalga *C. vulgaris*, both in their commercial medicine forms and as APIs. These results emphasize the importance of considering not only the isolated active substance but also the modulatory effects of excipients present in pharmaceutical medicines.^46^

Among the compounds tested, DTG-API showed the highest toxicity, in both acute and chronic exposures (EC50 = 3.3 mg/L and 5.9 mg/L, respectively), being the only one classified as “toxic” under both conditions. This high toxicity may be related to its moderately hydrophobic structure (log Kow = 1.6), which favors interaction with cell membranes, and its low water solubility, which may enhance its accumulation in algal cells.^62^

Moreover, the use of DMSO as a solvent may have facilitated medicine penetration into the cell. Although used at a low concentration (0.1%), which was not toxic on its own, it may have altered algal membrane permeability.^63^ In contrast, DTG-Med was less toxic under chronic exposure, suggesting that excipients may attenuate the active compound’s impact over time, possibly through complexation, adsorption, or reduced bioavailability.

For TDF, a distinct behavior was observed between commercial medicine and the API. While both were classified as “slightly toxic” or “practically nontoxic”, TDF-Med exhibited hormesis, with significant stimulation of algal growth at low chronic concentrations (EC50 for stimulation = 28.4 mg/L). This effect may be attributed to the activation of adaptive metabolic mechanisms in the microalga in response to low doses of the compound or its excipients, which may act as sources of carbon or nitrogen.^64^ On the other hand, TDF-API did not trigger such stimulation and showed greater chronic toxicity than acute, suggesting possible progressive bioaccumulation of the pure compound and the absence of modulating excipients.

In the case of 3TC, both the commercial medicine and the API demonstrated low overall toxicity, with EC50 values above 100.0 mg/L. Notably, 3TC-API induced chronic stimulation of algal growth, reinforcing the hypothesis that the substance could be used as an alternative nutrient or metabolic energy source by *C. vulgaris*.

Additionally, the absence of effects on the microalga’s metabolic activity is likely related to the medicine’s specific mechanism of action. 3TC is a nucleoside analog that inhibits reverse transcriptase, an enzyme absent in photosynthetic organisms. Therefore, its effects are likely limited to very high concentrations or nonspecific mechanisms. Gomes et al. (2022)^38^ calculated EC50 values for lamivudine above 570 mg/L for *S. elongatus* and *C. infusionum*, supporting the limited toxicity of 3TC on photosynthetic organisms.

Regarding metabolic activity, the most significant impacts were observed with DTG (especially the API), with reductions of up to 44%, even at concentrations that did not inhibit growth. This suggests the occurrence of sublethal effects, such as oxidative stress, impaired photosynthesis, or changes in respiration and protein synthesis cycles, before cellular proliferation is affected. The dissociation between lethal toxicity and metabolic effects underscores the importance of using multiple end points in ecotoxicological assessments.

Collectively, the data indicate that the effects of antiretrovirals on microalgae extend beyond growth inhibition, including adaptive responses,^65^ bioaccumulation,^54^ and possible interactions with excipients.^44^ This complexity highlights the risk of underestimating the ecological impact of pharmaceuticals when considering only isolated acute toxicity and emphasizes the need to include chronic exposures and sublethal metrics in regulatory protocols. The principal component analysis (PCA) represented in Figure 1 considers the acute EC50, chronic EC50, and metabolic activity variation of *C. vulgaris* as variables.

**Figure 1:**
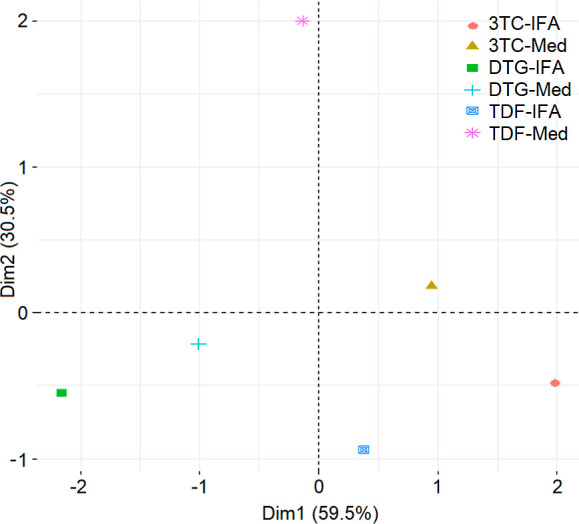
Principal component analysis (PCA) based on ecotoxicological parameters (acute EC50, chronic EC50, and metabolic activity variation) of the antiretrovirals dolutegravir (DTG), tenofovir (TDF), and lamivudine (3TC), in both commercial medicine (Med) and active pharmaceutical ingredient (API) forms, on *C. vulgaris*. Dim1 represents the first principal component, accounting for the largest proportion of the total data variance. It is derived from a standardized linear combination of acute EC50, chronic EC50, and metabolic activity, capturing the joint gradient in which these three parameters vary simultaneously. Dim2 corresponds to the second principal component, orthogonal to Dim1, and captures the second largest source of independent variation, highlighting patterns not explained by Dim1.

The PCA results revealed that DTG-API stood out from the other treatments, appearing isolated on the plot due to its high acute and chronic toxicity and marked reduction in metabolic activity. This separation indicates that DTG in its pure form has a unique toxicological profile, likely related to greater bioavailability and the absence of mitigating excipients. In contrast, 3TC-Med and 3TC-API clustered near the center of the plot, reinforcing their virtually nontoxic behavior and lack of significant metabolic impact.

Furthermore, the PCA showed that the first two components explained 90.0% of the total variance in the data, with 59.5% attributed to Component 1 (PC1) and 30.5% to Component 2 (PC2). PC1 was mainly associated with variations in acute and chronic EC50 values, reflecting the toxicity gradient among the evaluated antiretrovirals. Along this axis, DTG-API stood out, positioned separately due to its low EC50 values and high toxicity in both exposures. PC2, in turn, represented differences related to metabolic activity, allowing discrimination of compounds that caused marked sublethal effects (e.g., DTG-Med and TDF-Med) from those with minimal impact (e.g., 3TC-Med and 3TC-API).

Thus, the PCA showed that ecotoxicological patterns observed in *C. vulgaris* were determined not only by growth inhibition but also by sublethal responses, highlighting the relevance of multivariate analysis in understanding the integrated effects of antiretrovirals on aquatic photosynthetic organisms.

Another highlight was the position of TDF-Med, which also separated from the rest of the medicines, primarily due to its high chronic EC50 combined with a notable reduction in metabolic activity, along with the occurrence of hormetic effects at low concentrations. This pattern suggests an intermediate ecotoxicological profile, where sublethal effects coexist with low lethal toxicity. Finally, TDF-API was located between the extremes, behaving more similarly to 3TC but showing slight chronic toxicity.

Altogether, the multivariate analysis supports previous section findings and demonstrates that the toxicity of antiretrovirals to *C. vulgaris* depends not only on the active compound but also on the pharmaceutical form, presence of excipients, and the type of biological response considered (lethal vs sublethal).

The comparison between DTG-API and DTG-Med reveals a clear substance-type effect influenced by both formulation and exposure duration. In the short term exposure (4 days), DTG-API exhibited higher toxicity (EC50 = 3.3 ± 0.2 mg/L) compared to DTG-Med (EC50 = 7.1 ± 0.1 mg/L), suggesting that the presence of excipients in the commercial formulation may initially reduce bioavailability or delay uptake. However, this pattern shifts over longer exposure periods.

After 14 days, DTG-Med showed a marked decrease in apparent toxicity (EC50 > 100 mg/L), while DTG-API maintained consistent toxicity levels (EC50 = 5.9 ± 0.5 mg/L). This divergence indicates that formulation components can modulate exposure dynamics over time, potentially influencing dissolution, stability, and interaction with the test organism. Such differences highlight that the toxicological response cannot be attributed solely to the active compound, but rather to the combined effects of formulation and exposure duration.

From an environmental perspective, these findings underscore the importance of considering commercial formulations (Med) in ecotoxicological assessments. While APIs provide valuable insight into intrinsic toxicity, it is the formulated products that are ultimately released into aquatic environments.

The contrasting responses observed between DTG-API and DTG-Med suggest that excipients may either enhance or reduce bioavailability depending on exposure conditions, leading to variable toxicity outcomes. Therefore, prioritizing Med forms in environmental risk classification is essential to better reflect real-world exposure scenarios. Integrating formulation type with exposure duration provides a more realistic and robust framework for environmental risk assessment, supporting more accurate identification of compounds of concern.

### 3.5 Environmental Risk Assessment

ARVs can enter aquatic ecosystems through various pathways, including improper disposal of medicines, discharge of effluents from wastewater treatment plants, and human excretion, which contains unchanged residues or active metabolites of the medicines. Globally, the presence of these compounds has been widely reported across different environmental compartments. Table 5 presents the average, minimum, and maximum concentrations of the antiretrovirals lamivudine and tenofovir detected in the environment. Due to the scarcity of environmental data on DTG, it was not possible to conduct a comprehensive and accurate analysis of this medicines’s environmental impact on aquatic organisms.

**Table 5:** Measured Environmental Concentrations of Lamivudine (3TC) and Tenofovir (TDF), in μg/L, in Different Water Types Worldwide^26^, Obtained From the PHARMS-UBA Database.^a^

ARV	water type	ME*C*_mean_	ME*C*_min_	ME*C*_max_	RQ*
3TC	groundwater	0.010	0.008	0.011	<0.01
3TC	Leachate	0.188	0.020	0.400	<0.01
3TC	surface water—estuary	0.040	0.040	0.040	<0.01
3TC	surface water—river	31.361	0.016	228.300	0.26
3TC	surface water—other	7.840	2.470	13.650	0.06
3TC	WWTP effluent—treated	45.003	0.020	847.100	0.37
3TC	WWTP effluent—untreated	18.245	0.020	118.970	0.15
TDF	surface water—lake	0.110	0.110	0.110	<0.01
TDF	surface water—river	0.192	0.145	0.243	<0.01
TDF	WWTP effluent—treated	0.050	0.001	0.100	<0.01
TDF	WWTP effluent—untreated	0.144	0.100	0.250	<0.01

a Caption: ME*C*_mean_ = mean measured environmental concentration; ME*C*_min_ = minimum measured environmental concentration; ME*C*_max_ = maximum measured environmental concentration; WWTP = wastewater treatment plants; RQ* = Risk Quotient calculated as the ratio between the mean environmental concentration and the PNEC value obtained from the cellular growth inhibition assay. Data compiled and processed by the authors from PHARMS-UBA database.^26^

Based on the results of this study, the PNEC (Predicted No-Effect Concentration) value of 3TC for the microalga *C. vulgaris* is 118.8 μg/L. This result considers the lowest observed effect concentration (growth inhibition after 4-d of exposure) and includes a safety margin for unforeseen harmful effects. Therefore, considering the highest environmental concentrations measured in surface river waters as well as in wastewater treatment plant effluents, both treated and untreated, these waters present a high risk for this single-celled organisms, since the RQ values were greater than 1.0.

In addition, a detailed analysis focused on wastewater treatment plant effluents was conducted to identify potential high, moderate, and low environmental risk levels associated with the release of the ARV 3TC into the environment. Table 6 presents the risk information based on 3TC concentrations found in both treated and untreated effluents.

**Table 6:** Concentrations of the Antiretroviral 3TC in Wastewater Treatment Plant Effluents (Treated and Untreated), Obtained From the PHARMS-UBA Database,^26^ and Corresponding Environmental Risk Classifications (RQ)^a^

place	year	concentration (μg/L)	RQ	risk
effluent from wastewater treatment plants—untreated				
Kenya	2012	30.300	0.26	medium
Kenya	2012	60.680	0.51	medium
Kenya	2012	31.460	0.26	medium
Belgium	2013	0.507	<0.01	insignificant
United States	2016	0.566	<0.01	insignificant
United States	2016	0.020	<0.01	insignificant
United States	2016	0.207	<0.01	insignificant
United States	2016	0.584	<0.01	insignificant
United States	2016	0.866	0.01	low
Republic of Zambia	2016	118.970	1.00	high
South Africa	2016	20.900	0.18	medium
South Africa	2016	3.670	0.03	low
South Africa	2016	2.200	0.02	low
South Africa	2016	0.840	0.01	low
South Africa	2016	1.900	0.02	low
Effluent from wastewater treatment plants—treated				
Kenya	2010	1.000	0.01	low
Kenya	2010	0.900	0.01	low
Kenya	2012	19.900	0.17	medium
Kenya	2012	31.070	0.26	medium
Kenya	2012	29.870	0.25	medium
Kenya	2014	3.985	0.03	low
Kenya	2019	847.100	7.13	high
United States	2016	0.020	0.01	low
Republic of Zambia	2016	55.760	0.47	medium
South Africa	2016	0.130	<0.01	insignificant
Germany	2016	0.060	<0.01	insignificant
Germany	2016	0.030	<0.01	insignificant
Germany	2016	0.040	<0.01	insignificant
Germany	2016	0.020	<0.01	insignificant

a Caption: RQ = Risk Quotient. Data compiled and processed by the authors from PHARMS-UBA database.^26^

The evaluation of ARV concentrations in wastewater treatment plant effluents did not show significant differences between treated and untreated sample groups. In untreated effluents (*n* = 15), risks were predominantly classified as low (*n* = 5) and insignificant (*n* = 5), followed by moderate risk (*n* = 4), and one case of high risk (*n* = 1). In treated effluents (*n* = 14), a relatively similar distribution was observed, with the highest frequency of sampling points classified as insignificant risk (*n* = 5) and low risk (*n* = 4), along with four samples showing moderate risk and one case of high risk.

These results indicate that although wastewater treatment contributes to the reduction of antiretroviral concentrations, it does not fully eliminate ecotoxicological risks, as moderate and even high-risk values were detected in treated samples, although it is important to note that these risks may be mitigated in natural receiving environments due to dilution and other environmental processes. The presence of significant risks after the treatment process reinforces the hypothesis that conventional treatment systems are not fully effective in removing this compound.

For the ARV TDF, based on the results of this study, the PNEC value for the microalga *C. vulgaris* is 92.9 μg/L. This value considers the lowest observed effect (growth inhibition after 4-d of exposure), like the data presented for 3TC, and includes a safety margin for unforeseen harmful effects. Therefore, considering the highest measured environmental concentrations, none of the sampling points where TDF was detected present any risk to this single-celled organisms, since for all evaluated points, the RQ value was below 0.01.

However, it is important to emphasize that the absence of identified risk for *C. vulgaris* does not necessarily imply universal environmental safety, as different aquatic organisms may have varying sensitivities to TDF. Furthermore, factors such as chronic exposures, interactions with other medicines in complex effluent mixtures, and possible bioaccumulation are not fully addressed in an analysis based solely on PNEC. Thus, although results indicate that TDF does not pose a significant threat to the evaluated microalga at reported environmental concentrations, there remains a need to expand ecotoxicological studies to include multiple species, different trophic levels, and realistic environmental conditions, to obtain a more comprehensive risk assessment.

### 3.6 Comparative Sensitivity of Microalgae and Cyanobacteria to ARVs

The comparative analysis between the previously published data for the cyanobacterium *M. novacekii* ^18^ and the present results obtained for the microalga *C. vulgaris* reveals taxon-dependent differences in susceptibility to antiretroviral drugs. In the earlier study, *M. novacekii* exhibited measurable growth inhibition following exposure to TDF, 3TC, and DTG, with EC50 values of 130.7, 184.8, and 1.7 mg/L after 4 days of exposure, indicating different sensitivities to these compounds under the tested conditions.

On the other hand, considering the same test conditions, the current data set demonstrates greater variability in responses for *C. vulgaris*. After exposure to TDF, 3TC, and DTG, EC50 values of 92.9, 118.8, and 7.1 mg/L were observed. Thus, distinct patterns of growth inhibition were observed, where for TDF and 3TC the microalga *C. vulgaris* was more sensitive than the cyanobacterium *M. novacekii*, while for DTG the microalga showed greater resistance when compared to the cyanobacterium.

These interspecific differences likely reflect fundamental biological distinctions between prokaryotic cyanobacteria and eukaryotic microalgae, including cellular organization, metabolic pathways, membrane transport systems, and potential differences in intracellular targets of ARVs. The presence of a true nucleus and distinct organelles in *C. vulgaris* may influence compound uptake, biotransformation capacity, and recovery dynamics during extended exposure. Collectively, the integration of both studies indicates that ARV toxicity cannot be generalized across aquatic primary producers, underscoring the importance of cross-taxonomic assessments to improve ecological risk characterization.

## 4 Environmental Implications and Degradation Perspectives

ARVs are essential medications in the treatment of HIV, but their presence in aquatic environments raises considerable environmental concerns. After administration, these medications are eliminated, often in active or partially metabolized forms,^66^ and can be introduced into water bodies through domestic, industrial, or hospital effluents.^13^ Once ARVs enter the aquatic environment, they can be biodegraded by single-celled organisms; however, this process is often slow and incomplete, causing these compounds to remain at detectable levels for a long time.^6^

The biodegradation of ARVs is influenced by several factors, such as the chemical structure of the compound, environmental conditions (temperature, pH, and nutrient availability), and the presence of specific single-celled organisms that can degrade them.^67^ Research suggests that compounds such as TDF are resistant to microbial degradation.^68,69^ However, even the most degradable ARVs can generate degradation products that preserve their biological activity and ecotoxicological potential.^39^

The continued presence of ARVs in the aquatic environment is alarming, as it can result in bioaccumulation in aquatic organisms.^54^ Furthermore, the constant presence of ARVs can lead to the development of resistance in aquatic single-celled organisms, compromising the effectiveness of antimicrobial treatments. Therefore, it is essential to monitor the presence of these compounds in water bodies and develop efficient methods for their elimination, aiming to protect environmental and human health.^70^

ARVs and other pharmaceutical pollutants can now be removed from aquatic environments using algae-based technologies, which are sustainable alternatives. By utilizing the inherent abilities of microalgae to adsorb, bioaccumulate, and degrade pharmaceutical compounds, these methods offer a cost-effective and environmentally beneficial way to treat contaminated waters. ARVs can be eliminated by microalgae in a number of ways, including adsorption, which lowers the concentration of the compounds in water due to physical interactions with the surfaces of the algal cells; bio absorption, which enables ARVs to be absorbed and held within algal cells; and intracellular enzymatic degradation, which converts ARVs into less harmful products and lessens their toxicity to the environment.^67,70^

Additionally, microalgae are essential for eliminating nutrients like phosphorus and nitrogen that are frequently linked to ARVs in wastewater, which helps to both purify water and lessen eutrophication in aquatic environments. Utilizing natural biological processes, algae-based technologies are sustainable, highly effective at eliminating a variety of pollutants, and produce valuable biomass that can be converted into fertilizer, biofuels, or bioactive compounds.^71^

These systems also reduce the amount of hazardous sludge and other residues that are produced by traditional treatment techniques. High-rate algal ponds are one example of a system that has shown effectiveness in treating wastewater containing ARVs. To improve their operational and financial viability, however, issues with growing conditions, algal growth, and biomass management need to be resolved. All things considered, algae-based technologies offer a viable and sustainable approach to eliminating ARVs and other newly discovered pollutants from aquatic environments, and their advantages for the environment and the economy make more study and development necessary before they can be widely used.

## 5 Conclusions

The results presented demonstrate that the ARVs evaluated exert significant ecotoxicological effects on the microalga *C. vulgaris*, with responses ranging from growth inhibition to detectable metabolic alterations. These findings reinforce the need to include continuously used pharmaceuticals, such as those employed in HIV treatment, in broader environmental risk assessments, as their persistence and bioactivity potential represent emerging threats to aquatic ecosystems. By integrating acute and chronic toxicity data with environmental risk metrics, this study provides robust evidence to support public policy aimed at pharmaceutical waste management and the protection of aquatic biodiversity. Ultimately, the results highlight not only the scientific relevance of investigating emerging contaminants of concern but also the urgency of regulatory actions to mitigate their presence in the environment, ensuring the sustainability of water resources and the preservation of ecosystem services.

## Supplementary Material



## Notes

Supporting Information toxicological assessment data, EC50 values, GHS classifications, statistical modeling results, and dose–response curves for *C. vulgaris* exposed to dolutegravir, tenofovir disoproxil fumarate, and lamivudine APIs and commercial formulations (PDF)

## Abbreviations

3TC: Lamivudine
3TC-API: Active Pharmaceutical Ingredient Lamivudine
3TC-Med: Commercial medicine based on Lamivudine
API: Active Pharmaceutical Ingredient
ARV: Antiretrovirals
CAS: Chemical Abstracts Service
CNPq: National Council for Scientific and Technological Development
DMSO: Dimethyl sulfoxide
drc: Dose Response Curves
DTG: Dolutegravir
DTG-API: Active Pharmaceutical Ingredient Dolutegravir
DTG-Med: Commercial medicine based on Dolutegravir
EC50: Concentration of effect on 50% of organisms
ERA: Environmental Risk Assessment
FAPEMIG: Minas Gerais State Research Support Foundation
FUNED: Ezequiel Dias Foundation
FURP: Fundação para o Remédio Popular
HIV: Human Immunodeficiency Virus
Log KOW: Logarithm of the *n*-octanol–water partition coefficient
MEC: Measured Environmental Concentration
MECmax: Maximum measured environmental concentration
MECmean: Mean measured environmental concentration
MECmin: Minimum measured environmental concentration
Med: Medicine
MTT: 3-(4,5-dimethylthiazol-2-yl)-2,5-diphenylformazan, thiazolyl blue
n.o: Not Observed
NaCl: sodium chloride
NaOH: sodium hydroxide
NOEC: No Observed Effect Concentrations
OECD: Organisation for Economic Co-operation and Development
PCA: principal component analysis
pH: hydrogen potential
p*K*_a_: Logarithm of the acid dissociation constant
pKb: Logarithm of the base dissociation constant
PNEC: Predicted No Effect Concentration
PVDF: Polyvinylidene fluoride
RQ: risk quotients
TDF: Tenofovir Disoproxil Fumarate
TDF-API: Active Pharmaceutical Ingredient Tenofovir Disoproxil Fumarate
TDF-Med: Commercial medicine based on Tenofovir Disoproxil Fumarate
UFMG: Federal University of Minas Gerais
VAL: Valsartan
WWTP: Wastewater Treatment Plants
