# Supplementary material for: Toxicity of the Antiretrovirals Lamivudine, Dolutegravir, and Tenofovir on the Microalga Chlorella vulgaris
Source: ACS Omega. 2026 Jun 1;11(23):33729–42. doi: 10.1021/acsomega.6c00162 (PMC13280828; doi:10.1021/acsomega.6c00162)

Toxicity of the antiretrovirals lamivudine, dolutegravir, and tenofovir on the microalga *Chlorella vulgaris*

Gabriel Souza-Silva 1,, Mariângela D. Alcantara 1, Carolina P. de S. Moreira 2, Maria Clara V. M. Starling 3, Cléssius R. de Souza 1, Kenia P. Nunes 4, Cíntia A. de J. Pereira 5, Marcos P. G. Mol 2 and Micheline R. Silveira 1*

1 - Postgraduate Program in Medicines and Pharmaceutical Assistance, School of Pharmacy, Federal University of Minas Gerais, Belo Horizonte 31270-901, MG; Brazil; 2 - Department of Research and Development, Ezequiel Dias Foundation, Department of Research and Development, Belo Horizonte 30510-010, MG, Brazil; 3 - Department of Sanitary and Environmental Engineering, School of Engineering, Federal University of Minas Gerais, Belo Horizonte 31270-901, MG, Brazil; 4 - Department of Biomedical Engineering and Science, Florida Institute of Technology, Melbourne, FL 32901, USA; 5 - Department of Parasitology, Institute of Biological Sciences, Federal University of Minas Gerais, Belo Horizonte 31270-901, MG, Brazil.

Table S1. Toxicological assessment results of the active pharmaceutical ingredient (API) and commercial medicines based on dolutegravir (DTG) on the microalga *Chlorella vulgaris*.

Test substance	Time (days)	EC50*	Effect	Statistical Model	GHS Classification
DTG-Med	4	7.1 ± 0.1 (6.9 – 7.4) mg/L	Growth Inhibition	Weibull	Toxic
DTG-Med	14	> 100.0 mg/L	Growth Inhibition	log-normal	Practically non-toxic
DTG-Med	4	n.o	Growth Stimulation	Weibull	-
DTG-Med	14	n.o	Growth Stimulation	Weibull	-
DTG-Med	4	68.8 ± 3.8 (59.4 – 78.3) mg/L	Algaecide	log-normal	Slightly toxic
DTG-Med	14	> 100.0 mg/L	Algaecide	Weibull	Practically non-toxic
DTG-API	4	3.3 ± 0.2 (2.8 – 3.8) mg/L	Growth Inhibition	log-logistic	Toxic
DTG-API	14	5.9 ± 0.5 (4.7 – 7.1) mg/L	Growth Inhibition	log-normal	Toxic
DTG-API	4	n.o	Growth Stimulation	log-normal	-
DTG-API	14	n.o	Growth Stimulation	Weibull	-
DTG-API	4	28.2 ± 4.4 (17.3 – 39.1) mg/L	Algaecide	Weibull	Slightly toxic
DTG-API	14	36.2 ± 2.2 (30.7 – 41.7) mg/L	Algaecide	log-logistic	Slightly toxic

Caption: * = values given as mean ± standard deviation (95% confidence interval); DTG-API = active pharmaceutical ingredient dolutegravir; DTG-Med = commercial medicine based on dolutegravir; EC50 = concentration of effect on 50% of organisms; n.o = not observed. The notation "not observed" indicates that the specific target effect, whether Growth Inhibition, Growth Stimulation, or Algaecide, was not elicited by the compound within the tested concentration range, i.e., the substance did not produce the intended effect at any of the concentrations evaluated.

Table S2. Toxicological evaluation results of the active pharmaceutical ingredient (API) and the commercial medicine based on tenofovir disoproxil fumarate (TDF) on the microalga *C. vulgaris*.

Test substance	Time (days)	EC50*	Effect	Statistical Model	GHS Classification
TDF-Med	4	92.9 ± 2.7 (86.2 – 99.6) mg/L	Growth Inhibition	Weibull	Slightly toxic
TDF-Med	14	> 400.0 mg/L	Growth Inhibition	Weibull	Practically non-toxic
TDF-Med	4	n.o	Growth Stimulation	log-logistic	-
TDF-Med	14	28.4 ± 3.3 (20.2 – 36.6) mg/L	Growth Stimulation	log-normal	Slightly toxic
TDF-Med	4	n.o	Algaecide	log-normal	-
TDF-Med	14	n.o	Algaecide	Weibull	-
TDF-API	4	110.3 ± 5.3 (97.1 – 123.5) mg/L	Growth Inhibition	Weibull	Practically non-toxic

TDF-API	14	88.7 ± 9.8 (64.3 – 113.1) mg/L	Growth Inhibition	log-logistic	Slightly toxic
TDF-API	4	n.o	Growth Stimulation	log-logistic	-
TDF-API	14	n.o	Growth Stimulation	Weibull	-
TDF-API	4	n.o	Algaecide	log-logistic	-
TDF-API	14	n.o	Algaecide	Weibull	-

Caption: * = values given as mean ± standard deviation (95% confidence interval); TDF-API = active pharmaceutical ingredient tenofovir disoproxil fumarate; TDF-Med = commercial medicine based on tenofovir disoproxil fumarate; EC50 = concentration of effect on 50% of organisms; n.o = not observed. The notation "not observed" indicates that the specific target effect, whether Growth Inhibition, Growth Stimulation, or Algaecide, was not elicited by the compound within the tested concentration range, i.e., the substance did not produce the intended effect at any of the concentrations evaluated.

Table S3. Toxicological evaluation results of the active pharmaceutical ingredient (API) and commercial medicine based on lamivudine (3TC) on the microalga *C. vulgaris*.

Test substance	Time (days)	EC50*	Effect	Statistical Model	GHS Classification
3TC-Med	4	118.8 ± 4.2 (100.7 – 136.9) mg/L	Growth Inhibition	Weibull	Practically non-toxic

3TC-Med	14	209.6 ± 24.6 (103.7 – 315.5) mg/L	Growth Inhibition	log-normal	Practically non-toxic
3TC-Med	4	n.o	Growth Stimulation	log-normal	-
3TC-Med	14	n.o	Growth Stimulation	log-logistic	-
3TC-Med	4	n.o	Algaecide	Weibull	-
3TC-Med	14	n.o	Algaecide	log-logistic	-
3TC-API	4	> 400.0 mg/L	Growth Inhibition	log-normal	Practically non-toxic
3TC-API	14	> 400.0 mg/L	Growth Inhibition	log-normal	Practically non-toxic
3TC-API	4	n.o	Growth Stimulation	log-logistic	-
3TC-API	14	155.0 ± 8.5 (118.4 – 191.6) mg/L	Growth Stimulation	Weibull	Practically non-toxic
3TC-API	4	n.o	Algaecide	log-normal	-
3TC-API	14	n.o	Algaecide	log-logistic	-

Caption: * = values given as mean ± standard deviation (95% confidence interval); 3TC-API = active pharmaceutical ingredient lamivudine; 3TC-Med = commercial medicine based on lamivudine; EC50 = concentration of effect on 50% of organisms; n.o = not observed. The notation "not observed" indicates that the specific target effect, whether Growth Inhibition, Growth Stimulation, or Algaecide, was not elicited by the compound within the tested concentration range, i.e., the substance did not produce the intended effect at any of the concentrations evaluated.

Figure S1 - Dose–response curves of *C. vulgaris* growth inhibition by DTG-Med at concentrations ranging from 0.01 to 100 mg/L for 96 h under controlled temperature (22.0°C ±1.0 ◦C), photoperiod (12/12 h light/dark) and continuous stirring (140 rpm).

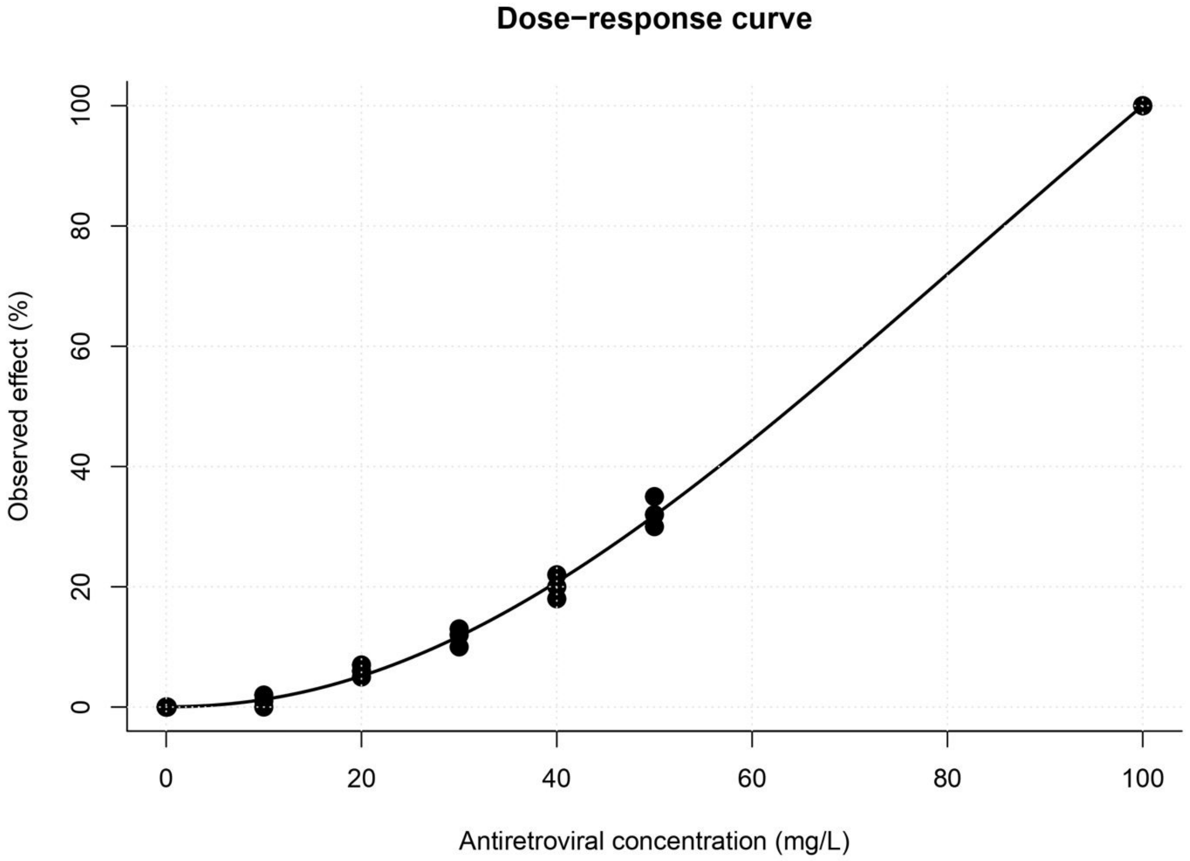

Figure S2 – Algacidal dose–response curves of *C. vulgaris* by DTG-Med at concentrations ranging from 0.01 to 100 mg/L for 96 h under controlled temperature (22.0°C ±1.0 ◦C), photoperiod (12/12 h light/dark) and continuous stirring (140 rpm).

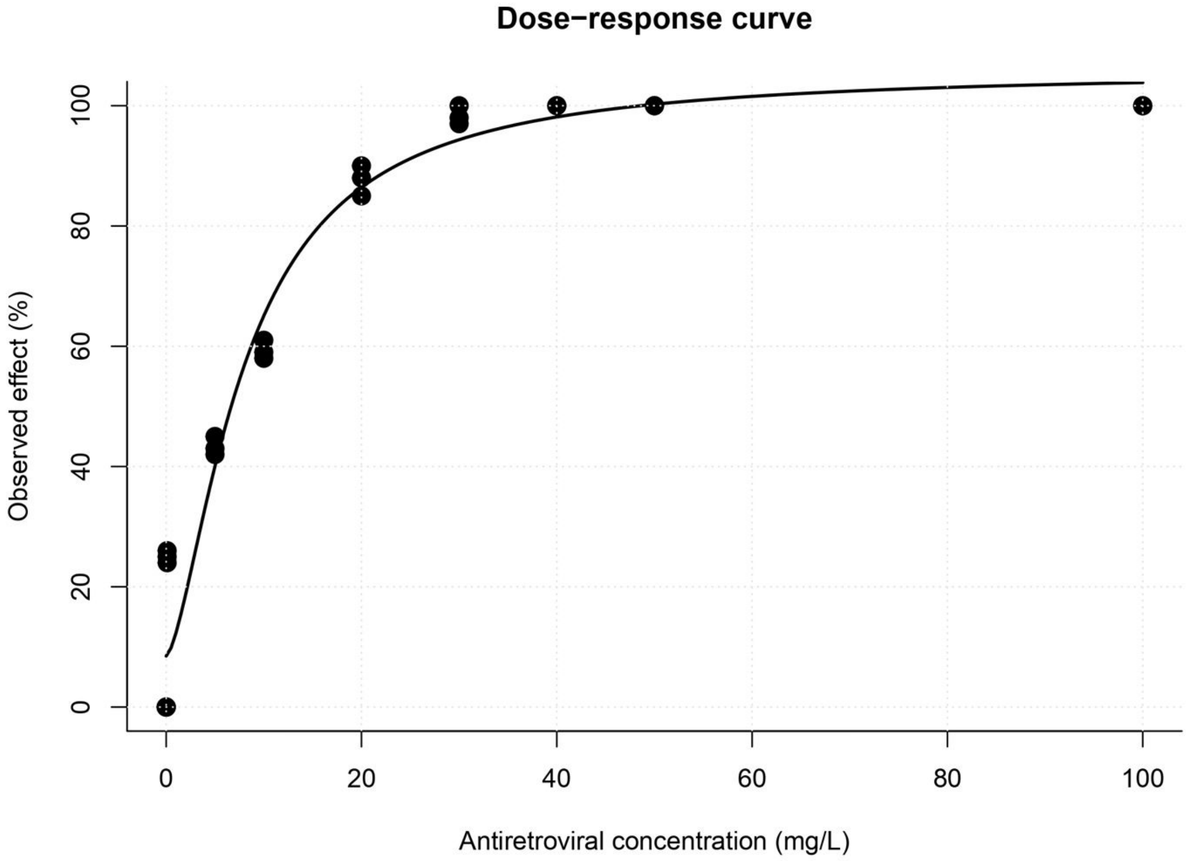

Figure S3 - Dose–response curves of *C. vulgaris* growth inhibition by DTG-API at concentrations ranging from 0.01 to 100 mg/L for 96 h under controlled temperature (22.0°C ±1.0 ◦C), photoperiod (12/12 h light/dark) and continuous stirring (140 rpm).

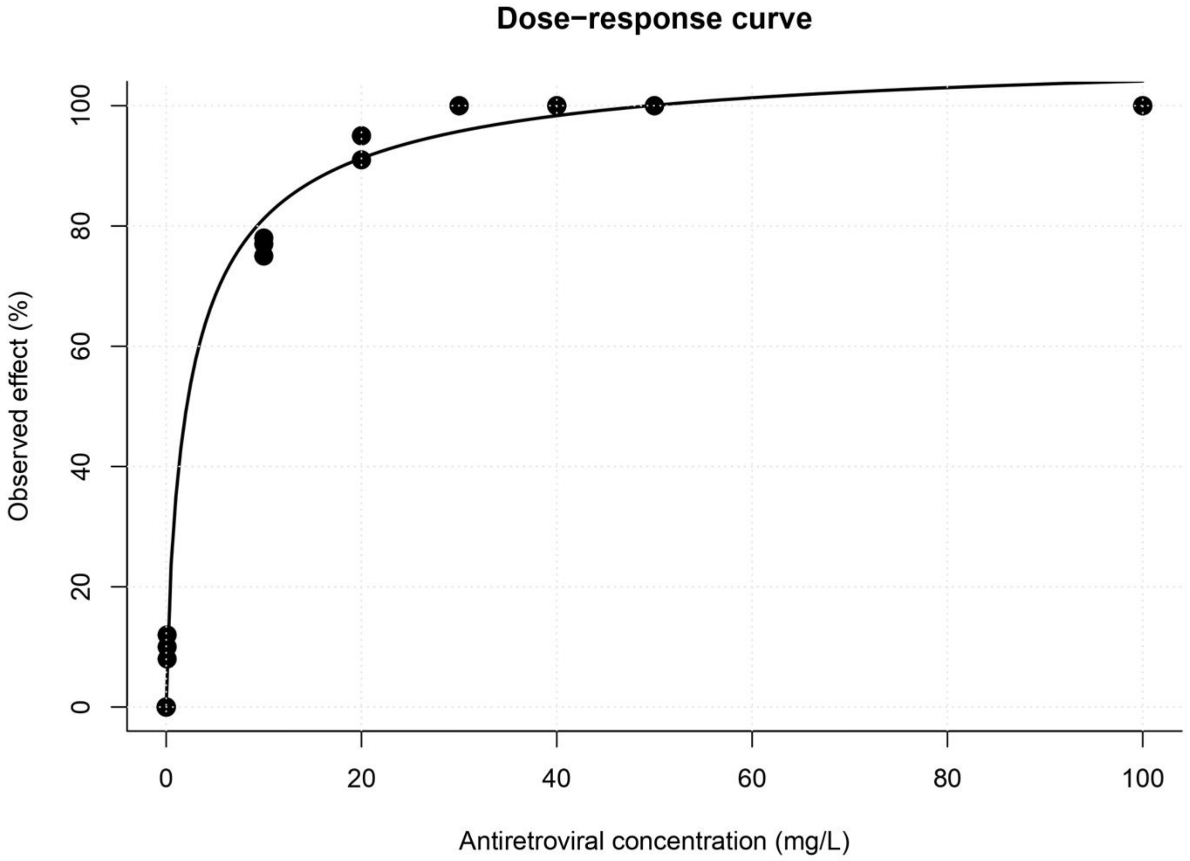

Figure S4 - Dose–response curves of *C. vulgaris* growth inhibition by DTG-API at concentrations ranging from 0.01 to 100 mg/L for 14 days under controlled temperature (22.0°C ±1.0 ◦C), photoperiod (12/12 h light/dark) and continuous stirring (140 rpm).

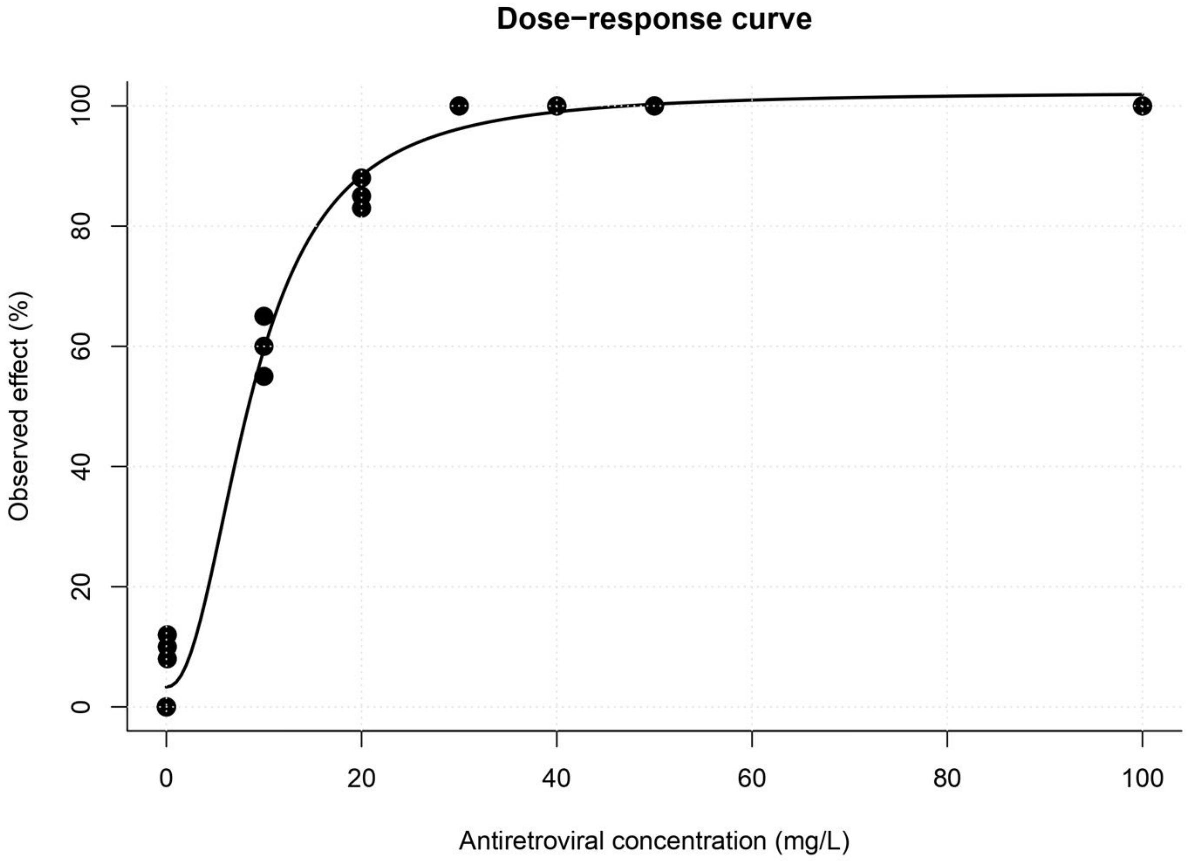

Figure S5 – Algacidal dose–response curves of *C. vulgaris* by DTG-API at concentrations ranging from 0.01 to 100 mg/L for 96 h under controlled temperature (22.0°C ±1.0 ◦C), photoperiod (12/12 h light/dark) and continuous stirring (140 rpm).

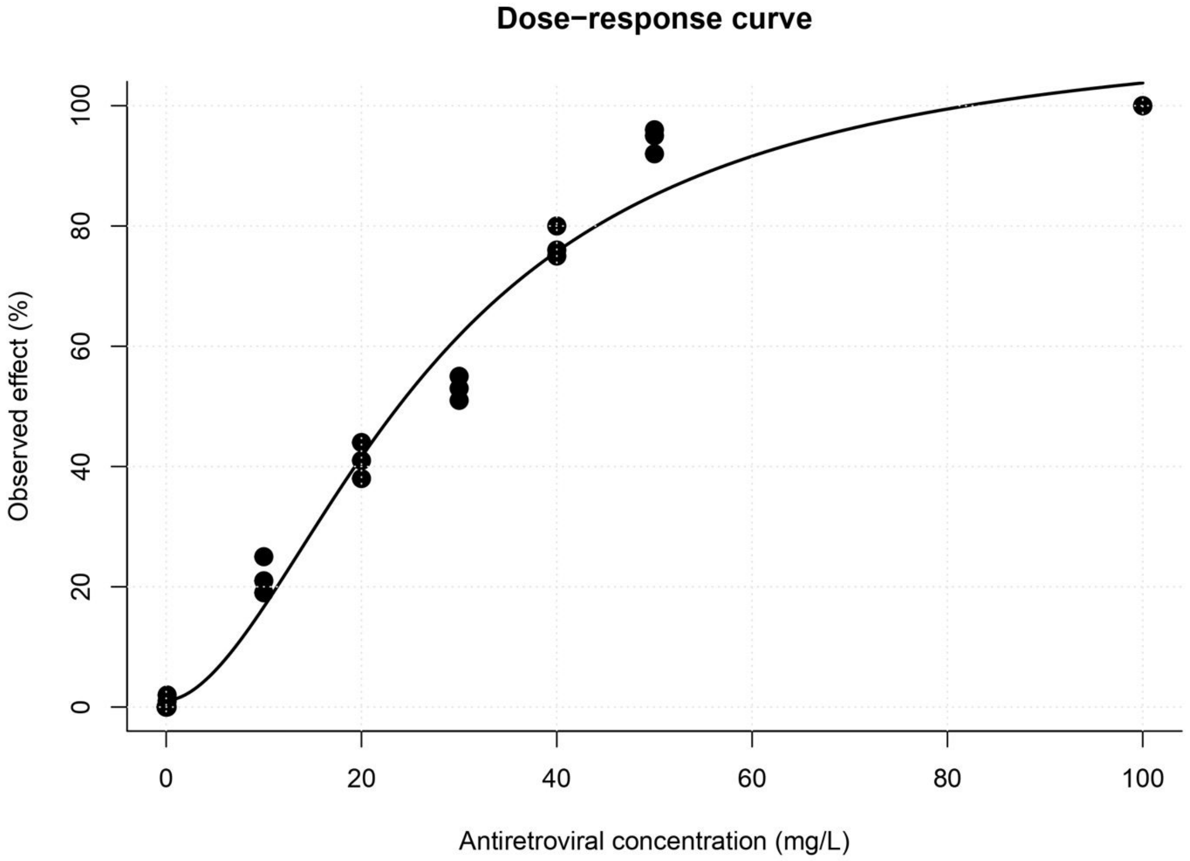

Figure S6 – Algacidal dose–response curves of *C. vulgaris* by DTG-API at concentrations ranging from 0.01 to 100 mg/L for 14 days under controlled temperature (22.0°C ±1.0 ◦C), photoperiod (12/12 h light/dark) and continuous stirring (140 rpm).

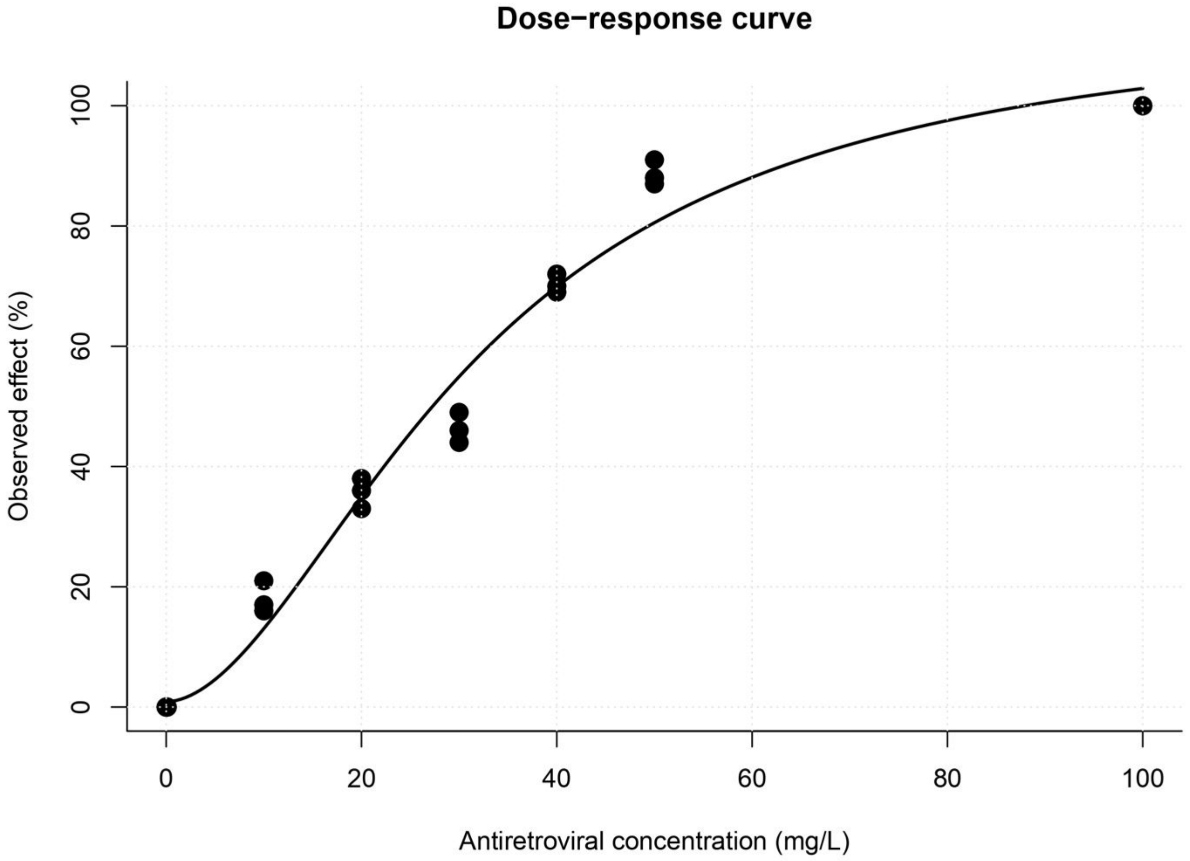

Figure S7 - Dose–response curves of *C. vulgaris* growth inhibition by TDF-Med at concentrations ranging from 0.01 to 100 mg/L for 96 h under controlled temperature (22.0°C ±1.0 ◦C), photoperiod (12/12 h light/dark) and continuous stirring (140 rpm).

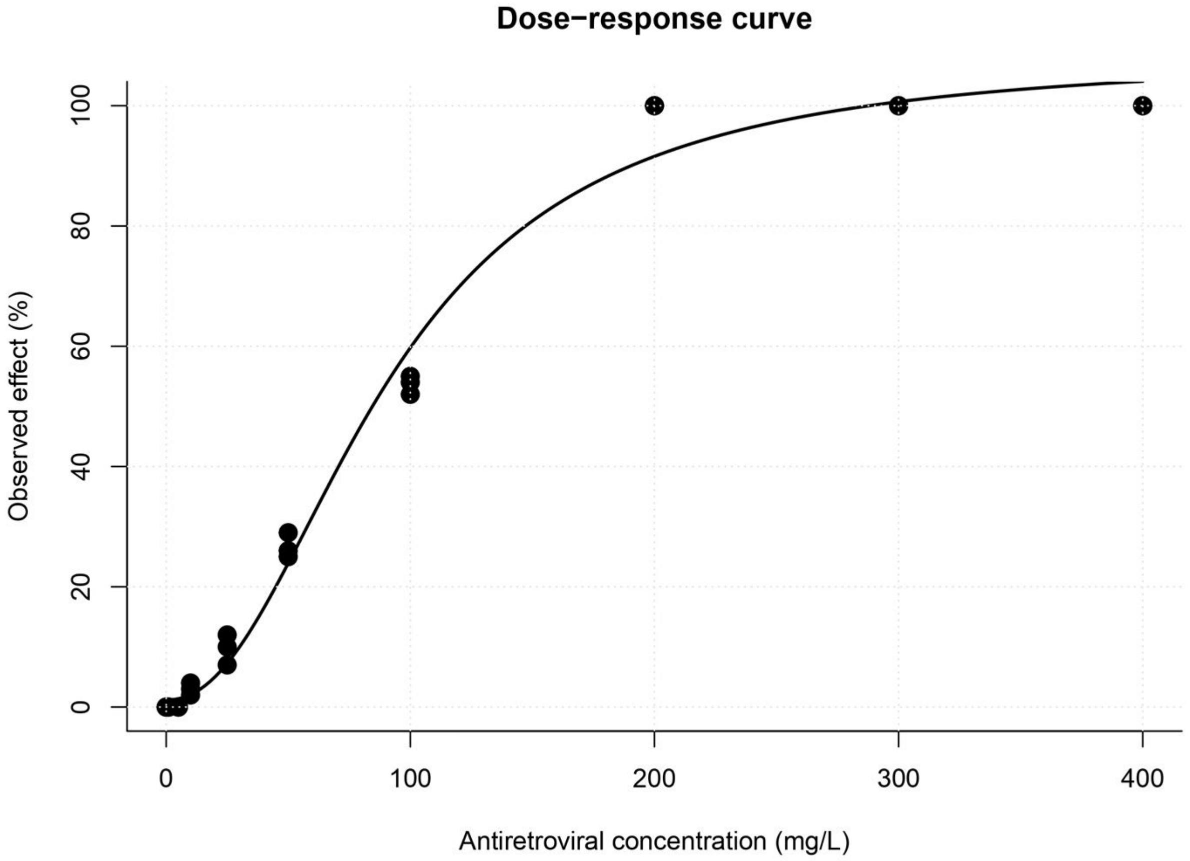

Figure S8 - Dose–response curves of *C. vulgaris* growth stimulation by TDF-Med at concentrations ranging from 0.01 to 100 mg/L for 14 days under controlled temperature (22.0°C ±1.0 ◦C), photoperiod (12/12 h light/dark) and continuous stirring (140 rpm).

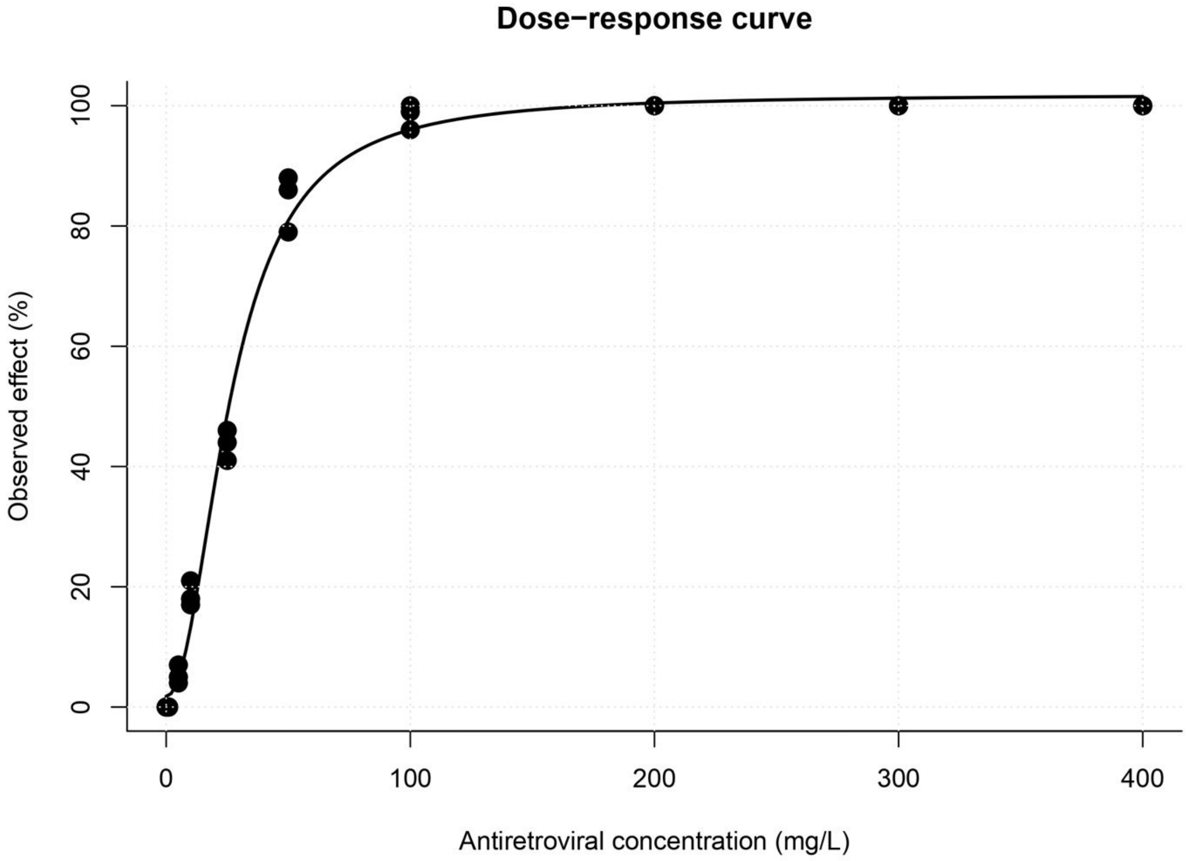

Figure S9 - Dose–response curves of *C. vulgaris* growth inhibition by TDF-API at concentrations ranging from 0.01 to 100 mg/L for 96 h under controlled temperature (22.0°C ±1.0 ◦C), photoperiod (12/12 h light/dark) and continuous stirring (140 rpm).

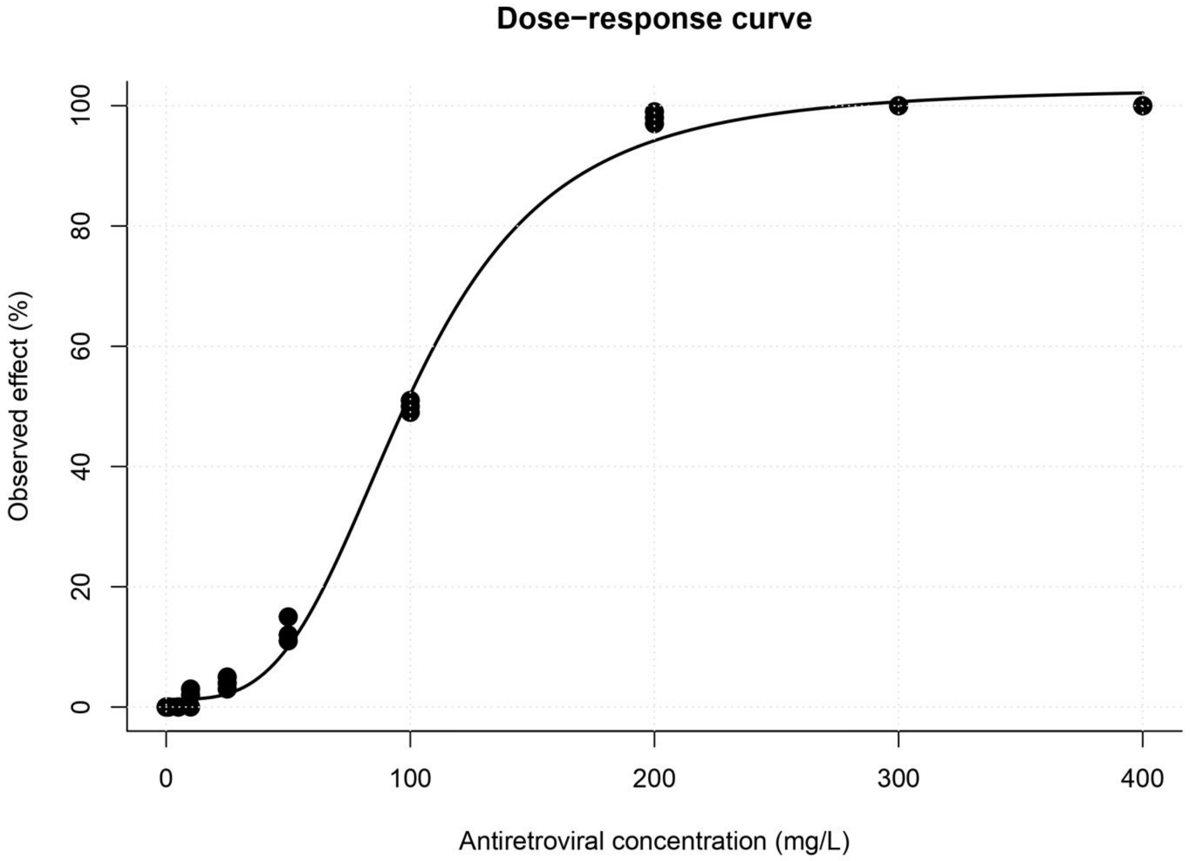

Figure S10 - Dose–response curves of *C. vulgaris* growth inhibition by TDF-API at concentrations ranging from 0.01 to 100 mg/L for 14 days under controlled temperature (22.0°C ±1.0 ◦C), photoperiod (12/12 h light/dark) and continuous stirring (140 rpm).

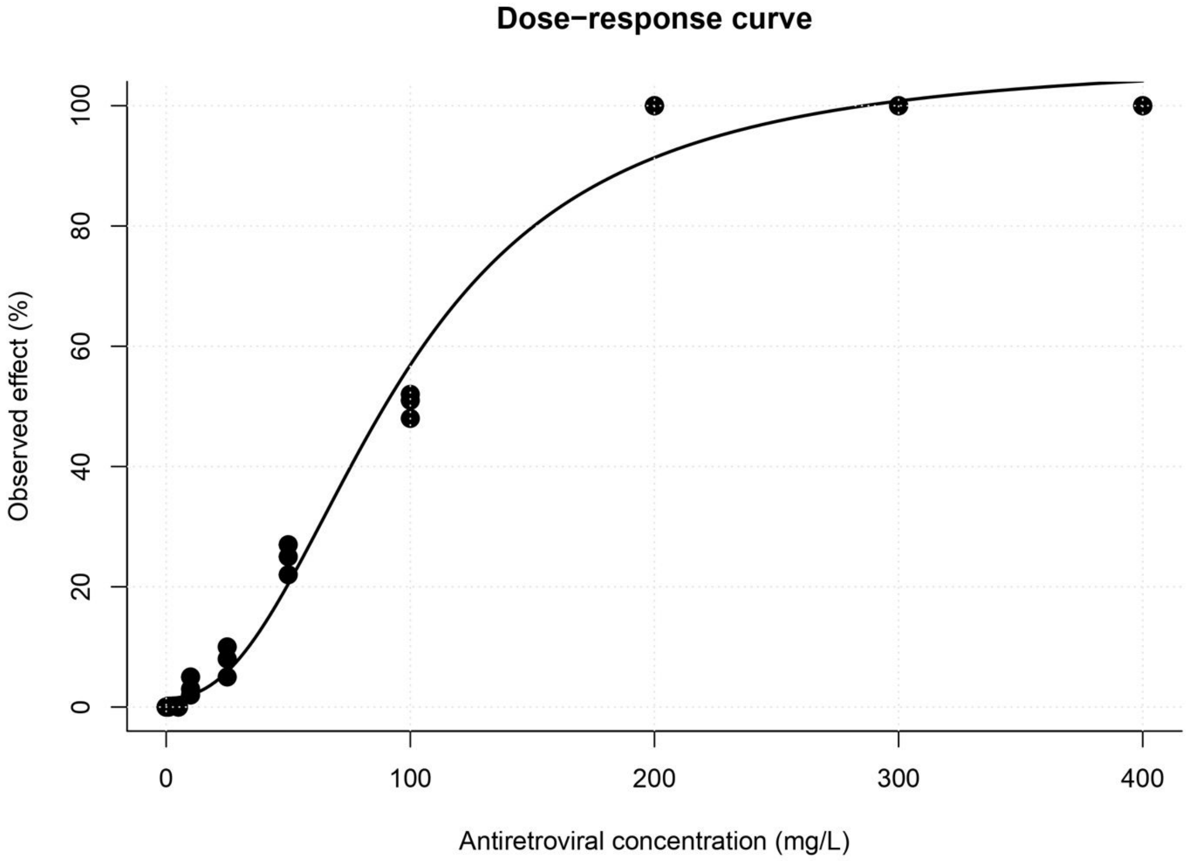

Figure S11 - Dose–response curves of *C. vulgaris* growth inhibition by 3TC-Med at concentrations ranging from 0.01 to 100 mg/L for 96 h under controlled temperature (22.0°C ±1.0 ◦C), photoperiod (12/12 h light/dark) and continuous stirring (140 rpm).

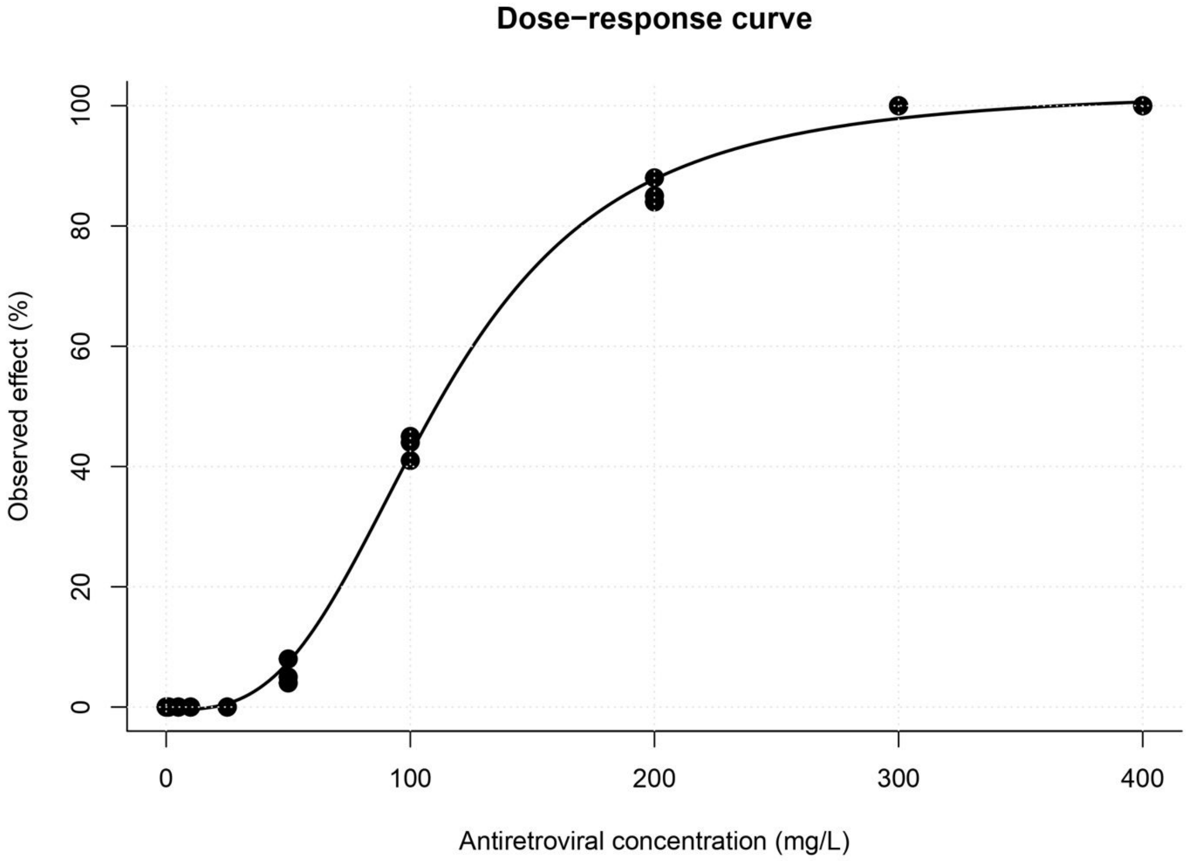

Figure S12 - Dose–response curves of *C. vulgaris* growth inhibition by 3TC-Med at concentrations ranging from 0.01 to 100 mg/L for 14 days under controlled temperature (22.0°C ±1.0 ◦C), photoperiod (12/12 h light/dark) and continuous stirring (140 rpm).

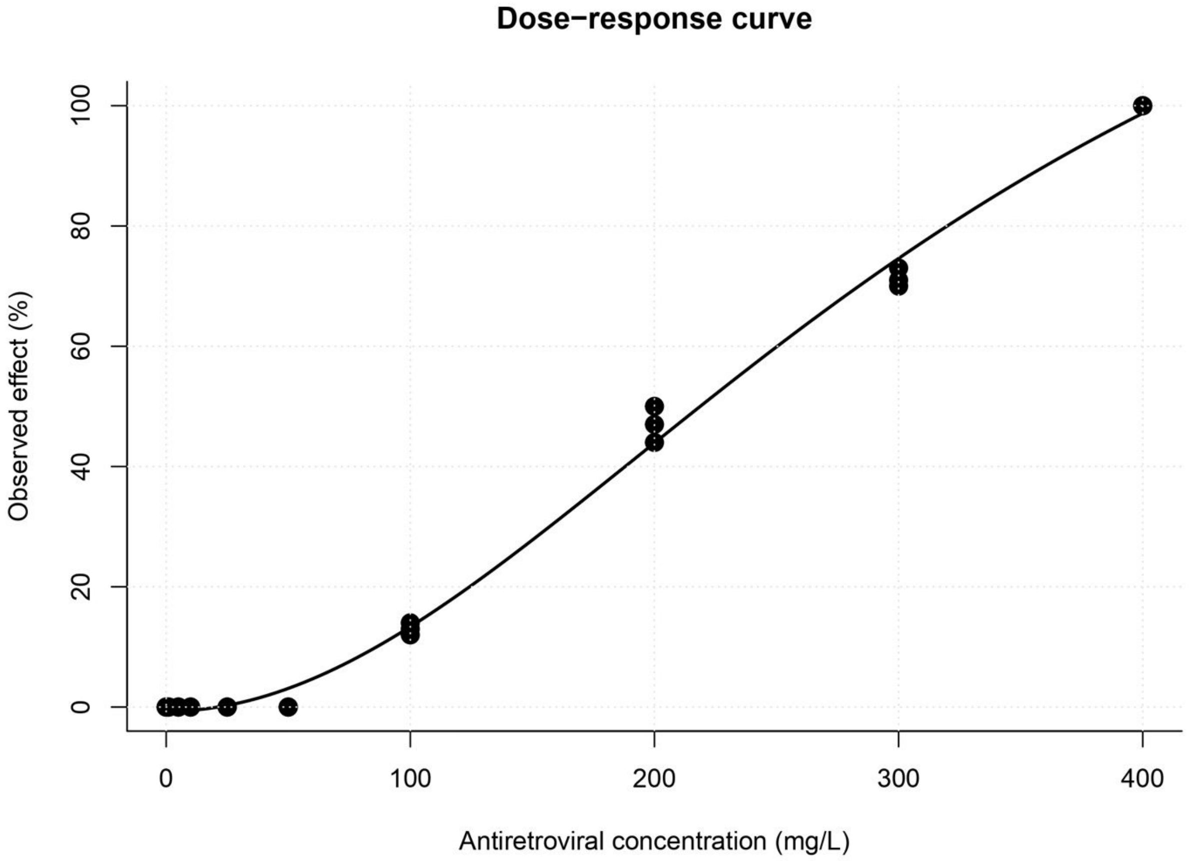

Figure S13 - Dose–response curves of *C. vulgaris* growth stimulation by 3TC-Med at concentrations ranging from 0.01 to 100 mg/L for 14 days under controlled temperature (22.0°C ±1.0 ◦C), photoperiod (12/12 h light/dark) and continuous stirring (140 rpm).

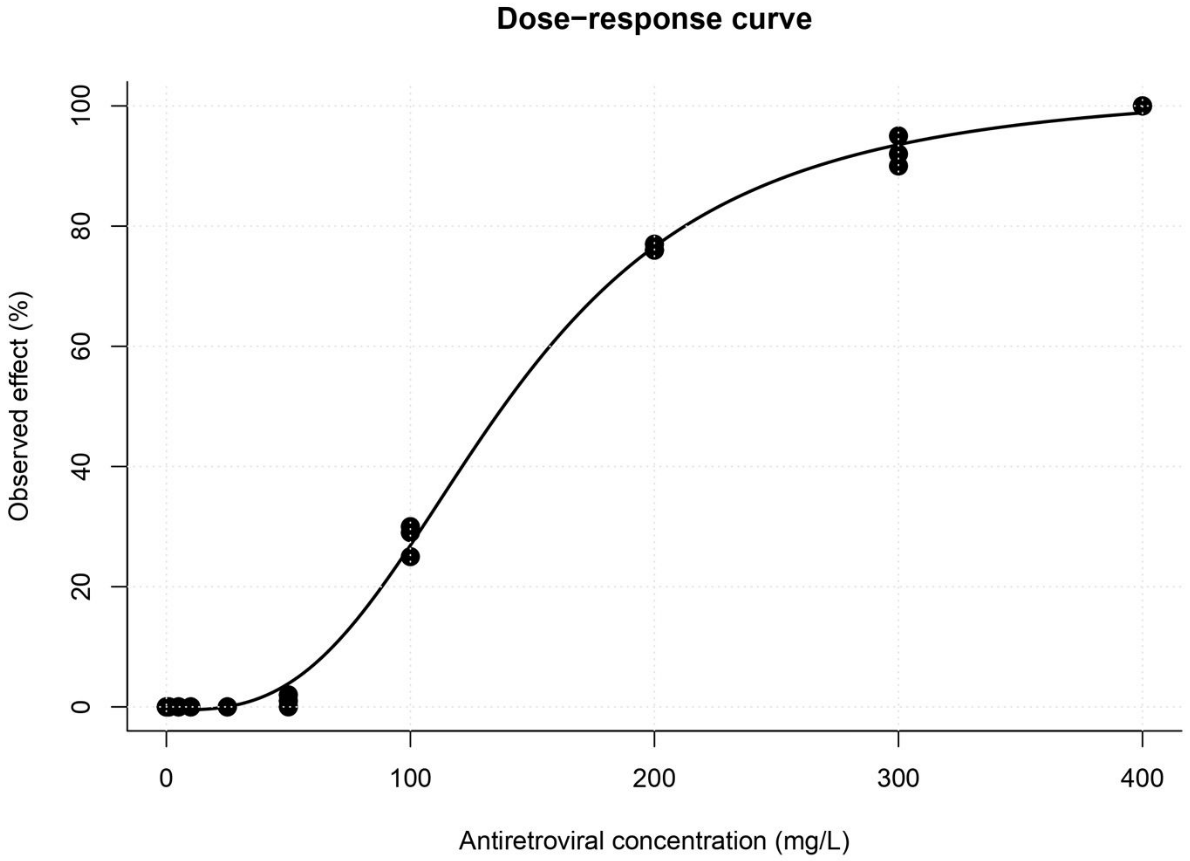

Supplement: Supplementary file 1 [file ao6c00162_si_001.pdf]
